# Zebrafish Agr2 Is Required for Terminal Differentiation of Intestinal Goblet Cells

**DOI:** 10.1371/journal.pone.0034408

**Published:** 2012-04-13

**Authors:** Yi-Chung Chen, Yu-Fen Lu, I-Chen Li, Sheng-Ping L. Hwang

**Affiliations:** 1 Institute of Cellular and Organismic Biology (ICOB), Academia Sinica, Taipei, Taiwan, Republic of China; 2 Institute of Molecular and Cellular Biology, National Tsing Hua University, Hsinchu, Taiwan, Republic of China; Cincinnati Children's Hospital Medical Center, United States of America

## Abstract

**Background:**

Mammalian Anterior Gradient 2 (AGR2) is a protein disulfide isomerase that is required for the production of intestinal mucus and Paneth and goblet cell homeostasis. However, whether increased endoplasmic reticulum (ER) stress occurs in *Agr2^−/−^* mice remains a controversial issue.

**Methodology/Principal Findings:**

We characterized the function of zebrafish *agr2* by both morpholino antisense oligomer-mediated knockdown and *agr2* mRNA overexpression. Fluorescent whole-mount double *in situ* hybridization indicated that in the intestine, *agr2* was only expressed in goblet cells. Significantly increased numbers of immature Alcian blue-stained goblet cells were observed in the intestines of 104- and 120-hours post fertilization (hpf) *agr2* morphants. Transmission electron microscopy analyses further confirmed the existence of immature pre-goblet cells containing few mucous granules in the mid-intestines of 104- and 120-hpf *agr2* morphants. *agr2* expression was not significantly induced by an ER stress inducer, tunicamycin. Expression of the ER chaperone gene *hspa5*, the spliced form of *xbp1s*, c/enhancer binding protein homologous protein *chop*, and the activating transcription factor 4b1 *atf4b1* were not significantly induced in either 104-hpf *agr2* morphants or *agr2*-overexpressed embryos. Similar percentages of P-Histone H3-stained M phase cells were identified in intestines of 104-hpf *agr2* morphants and control embryos.

**Conclusions/Significance:**

Our study demonstrates that in contrast to mouse AGR2, zebrafish Agr2 is expressed in only one intestinal secretory cell type - the goblet cells. Agr2 is essential for terminal differentiation of intestinal goblet cells in zebrafish embryos. Either knockdown of *agr2* function or *agr2* overexpression could not extensively induce expression of members of the unfolded protein response pathway.

## Introduction

*Anterior gradient 2* genes, such as *XAG-1* and *XAG-2*, were first identified as cement gland-specific genes in *Xenopus* [1]. Studies have shown that the function of XAG-2 in ectodermal patterning is to specify the differentiation of the cement gland and forebrain [2]. Subsequently, *anterior gradient 2* homologues have been identified in different vertebrates ranging from newts to mammals. Newt nAG was shown to play an essential role in the regeneration of the limb [3]. Two human homologues (*HAGR2* and *HAGR3*) have been identified. They are located adjacent to one another on chromosome 7, and they share 71% amino acid sequence similarity [4]. Activated *AGR2* expression was found in different human cancer cells including breast, prostate, ovarian, esophagus, gastro-intestinal tract and lung, indicating a role in promoting cell proliferation [5]. Knockdown of *AGR2* expression in estrogen receptor-α-positive breast cancer cell lines inhibited cell growth and induced cell death by modulating expression of *cyclin D1*, estrogen receptor-α, and *survivin* [6]. Recent work demonstrated that human AGR2 promotes cell growth by modulating expression of *amphiregulin*, an EGFR ligand, and *YAP1*, a co-activator of the HIPPO pathway [7]. Furthermore, human AGR2 was considered to be a metastasis inducer because high incidences of metastases occurred in the lungs of animals receiving AGR2 transfectants [8].

Human AGR2 and AGR3 were later identified as novel members of the protein disulfide isomerase family [9]. They contain one thioredoxin (Tx)-domain motif (CXXC), which facilitates oxidation and reduction. HAGR2 and HAGR3 shared high amino acid similarity with ERp18/19 and contain reminiscence of an ER retrieval signal in their C-termini (KTEL and QSEL, respectively). The mouse homolog of *Agr2*, *GOB-4*, was shown to be strongly expressed in the adult small intestine, colon, and stomach [10]. Immunohistochemistry further demonstrated that mouse AGR2 was localized in proliferating intestinal stem cells/early progenitor cells (such as Musashi-1 (MSI1)-positive cells at the crypt base) and also in differentiated secretory cell lineages, including goblet, Paneth and enteroendocrine cells [11]. Both germline and inducible *Agr2* ^−/−^ mice were generated by two different studies to investigate AGR2 function in intestinal development [12], [13]. Mouse AGR2 was demonstrated to localize to the lumen of the endoplasmic reticulum (ER) of intestinal secretory epithelial cells and is required for the production of mucin and mucus in the intestine [13]. Mouse AGR2 was also shown to be required to maintain homeostasis of both Paneth and goblet cells in the intestine [12]. Furthermore, mice lacking *Agr2* were susceptible to colitis, suggesting a role in the protection from diseases such as inflammatory bowel disease. The results from mouse models and human inflammatory bowel disease demonstrated a close relationship between ER stress, activation of the unfolded protein response (UPR) and intestinal inflammation [14]. However, different results regarding whether ER stress is induced in the intestine of different *Agr2^−/−^* null mice were reported [12], [13].

Zebrafish has been widely used as an important model organism for the study of gastrointestinal development and related human diseases [15]. Compared to the mammalian intestinal epithelium, zebrafish do not have either crypts of Lieberkuhn or Paneth cells. Zebrafish villi possess three different differentiated cell types, these include enterocytes, which are responsible for nutrient absorption; goblet cells, which secrete the mucus layer to protect the intestinal epithelium from pathogens; and enteroendocrine cells, which produce different hormones that maintain normal physiological function [16]. Previously, we cloned and characterized the zebrafish *agr2* gene [17]. Whole-mount *in situ* hybridization demonstrated that *agr2* is expressed in most organs that contain mucus-secreting cells, including epidermis, olfactory bulbs, otic vesicles, pharynx, esophagus, pneumatic duct, swim bladder, and intestine. In this study, both morpholino antisense oligomer knockdown and overexpression approaches were used to investigate *agr2* function in intestinal development. Knockdown of *agr2* expression caused defects in the maturation of intestinal goblet cells detected by both Alcian blue staining and transmission electron microscopy analysis. Either knockdown of *agr2* function or *agr2* overexpression could not extensively induce expression of members of the UPR pathway. Agr2 was not required for normal intestinal cell proliferation.

## Materials and Methods

### Zebrafish Maintenance and Staging

Wild type zebrafish AB strain was maintained as previously described [18]. Different developmental stages were determined as described [19]. All animal procedures were approved by the Animal Use and Care Committee of Academia Sinica (protocol # RFiZOOHS2009095).

### Morpholino Antisense Oligomer-Mediated Knockdown Analysis

Two translational morpholino oligomers (MOs) (Gene Tools) were designed to prevent *agr2* protein synthesis. Their sequences were as follows: agr2-MO1, comprising sequences complementary to the AUG translational start site and the 22 bases in the 5′UTR region: GCACTGACAGTAATCCTTTGAGCAT ; agr2-MO2: AGCATCTTGATGAGCTGTGCTGAAT. Two control MOs were designed: agr2–5 mmMO1: GCAgTcACAcTAATCgTTTcAGCAT ; and agr2–5 mmMO2: AGCATgTTcATGAGgTGTcCTcAAT. Different diluted MOs were microinjected into the cytoplasm of 1–2 cell zygotes using a Nanoject II automatic injector (Drummond).

To demonstrate the specificity of two *agr2* translational MOs, we fused the *agr2* MO sequence in front of the *GFP* start codon which is under the control of *CMV* enhancer/promoter. GFP expression patterns were examined for embryos that had been respectively injected with 100 pg of *CMV*-*agr2 mo*-*GFP* plasmid alone, coinjected with 3.9 ng each of agr2-MO1 and agr2-MO2 and 100 pg of *CMV*-*agr2 mo*-*GFP* plasmid or with 3.9 ng each of agr2–5 mmMO1 and agr2–5 mmMO2 and 100 pg of *CMV*-*agr2 mo*-*GFP* plasmid at 30 hpf.

### Whole-Mount *In Situ* Hybridization, Fluorescent Whole-Mount Double *In Situ* Hybridization, Whole-Mount Immunohistochemistry, p-Histone H3 Immunostaining and Alcian Blue Staining

Whole-mount *in situ* hybridization was conducted on embryos treated with 0.003% phenylthiocarbamide using digoxigenin-labeled antisense RNA probes and alkaline phosphatase-conjugated anti-digoxigenin antibodies as described previously [20].

For fluorescent whole-mount double *in situ* hybridization, fixed and dehydrated embryos were incubated with 2% H_2_O_2_ in methanol for 20 min before rehydration and rinses in PBST (0.1% tween 20). After 40 min proteinase K (10 µg/ml) treatment, 20 min 4% paraformaldehyde fixation, and PBST washes, embryos were incubated in hybridization solution containing 5% (V/V) dextran sulfate and either fluorescein- or digoxigenin-labeled RNA probes at 65°C overnight. The next day, embryos were incubated in 5% serum for 1 h after RNA probe removal and washes. Embryos were then incubated with pre-absorbed horseradish peroxidase-conjugated anti-fluorescein antibody (1∶500) diluted in 5% serum at 4°C for overnight. The next day, embryos were washed with PBST and rinsed with a solution containing 100 mM borate, pH 8.6 and 0.1% tween 20 and then incubated with reaction buffer (100 mM borate, pH 8.6, 2% (V/V) dextran sulfate, 450 µg/ml 4-iodophenol, 0.1% tween 20, 0.003% H_2_O_2_) containing TSA plus fluorescein reagent (1∶100, Perkin Elmer) for 40 min. Embryos were then washed with PBST and the peroxidase reaction was inactivated by incubation in solution containing 100 mM glycine, pH 2.0 and 0.1% tween 20 for 10 min. Embryos were then blocked with 5% serum before incubation with horseradish peroxidase conjugated anti-digoxigenin antibody (1∶500) at 4°C overnight. The next day, embryos were stained with reaction solution containing TSA plus Cyanine 3 reagent (1∶100) for 40 min. Embryos were then washed with PBST, 50% methanol and 100% methanol, and incubated in 1% H_2_O_2_ in methanol for 30 min before fixation in 4% paraformaldehyde. Embryos were then stored in 80% glycerol. Two templates were linearized, and antisense RNA probes were produced as follows: *agr2* (*Bam* HI/T7) and *glucagon* (*Nco* I/SP6).

For whole-mount immunohistochemistry, embryos were fixed with 4% paraformaldehyde at 4°C for 3 h and then washed with PBS containing 0.1% triton x-100 and methanol. After rehydration, embryos were treated with proteinase K (10 µg/ml) for 40 min. Following PBST washes, embryos were incubated in 10% serum at room temperature for 1 h before incubation with anti-salmon Agr2 antiserum diluted in blocking solution containing 5% serum and 2 mg/ml BSA (1∶200) at 4°C overnight [21]. After PBST washes, embryos were incubated with Alexa 488-conjugated anti-rabbit secondary antibody (1∶200) at room temperature for 3 h. Embryos were stored in 80% glycerol following PBST washes.

Whole-mount Alcian blue staining of *agr2* morphants and agr2–5 mmMO1 and 5 mmMO2-coinjected, *agr2*-overexpressed, *lacZ*-overexpressed, and wild type embryos were performed as described [18]. p-Histone H3 immunostaining on *agr2* morphants and control embryos were carried out as described [18]. The percentage of p-Histone H3-stained cells was calculated by counting p-Histone H3-stained cell numbers and the total intestinal epithelial cell numbers from 6–10 sections of the mid-intestines and posterior intestines of 104-hpf *agr2* morphants, agr2–5 mmMO1 and 5 mmMO2-coinjected embryos and wild type embryos.

### Overexpression, Total RNA Extraction, Tunicamycin Treatment, and Real-Time Quantitative Reverse-Transcription-Polymerase Chain Reaction (qPCR)

Capped *agr2* and *lacZ*, mRNAs were synthesized using a T7 or a SP6 mMESSAGE mMACHINE Kit (Ambion). To overexpress *agr2*, *agr2* mRNA (200–250 pg) was injected into the cytoplasm of 1–2 cell zygotes. As a control, *lacZ* mRNA (250 pg) was injected the same way. Total RNA was extracted using RNeasy Plant Mini Kit (Qiagen).

For tunicamycin treatment, 96-hpf wild type embryos were incubated in respective 1, 2 or 3 µg/ml of tunicamycin in DMSO for 24 h and harvested at 120 hpf for total RNA extraction. As a control, 96-hpf wild type embryos were treated with DMSO (5 µg/ml) for 24 h and total RNA were harvested at 120 hpf.

To synthesize 1^st^-strand cDNA, a reaction buffer (15 µl) containing 1 µg total RNA and 500 ng random primer was heated at 70°C for 5 min and then chilled on ice. Reverse transcription reaction was then conducted by addition of 15 µl solution containing RT buffer (1× final concentration), MgCl_2_ (2.5 mM final concentration), 1 µl RNase inhibitor, dNTP (0.5 mM final concentration), and 3 µl Impron II Reverse Transcriptase (Promega). Reverse transcription reaction was incubated at 25°C for 10 min, 55°C for 1 h and then heated at 70°C for 10 min. qPCR was conducted in a 20 µl reaction buffer containing 83.3 ng diluted cDNA, forward and reverse primer pair (each of 0.25 µM final concentration), and SYBR Green I (1× final concentration) using a Roche Light Cycler 480 II machine. PCR cycles are: 95°C 10 min; 95°C 10 sec, 60°C 10 sec, 72°C 10 sec for 45 cycles; 95°C 5 sec; 65°C 1 min; 4°C 30 sec. The *agr2* primer pair was F: GGTCACGATCCAAGAACAAG and R: CCATCAGGAGACAAGTGCTT. The *hspa5* primer pair was F: CGAAGAAGCCAGATATCGATGA and R: ACGGCTCTTTTCCGTTGAAG. The *xbp1s* primer pair was F: TGTTGCGAGACAAGACGA and R: CCTGCACCTGCT GCGGACT. The *atf4b1* primer pair was F: CTTTCTCTCCTCCTGCTTCT and R: GAG TCACACGACCCAATCA. The *chop* primer pair was F: GAGTTGGAGGCGTGGTATGA and R: CCTTGGTGGCGATTGGTGAA. The *pdia5* primer pair was F: GGAGACGTAGGAGACTGGT and R: CACATCAGACAGCAGCTT. The *EF-1α* primer pair was F: CTGGAGGCCAGCTCAAACAT and R: ATCAAGAGTAGTACCGCTAGCATTAC.

The *β-actin* primer pair was F: CGAGCAGGAGATGGGAACC and R: GGGCAACGGAAACGCTCAT.

### Histology, Photography, and Electron Microscopy

Cryostat sectioning of 104-hpf *agr2* morphants, agr2–5 mmMO1 and 5 mmMO2-coinjected, and wild type embryos were performed as described [22]. Images of embryos were taken using either an RT color digital camera (SPOT) on a Zeiss Axioplan 2 microscope equipped with DIC mode or a Leica DFC 310 FX on a Leica Z16 Apo stereomicroscope. Fluorescent images were taken using a Leica TCS-SP5-MP confocal microscope. Transmission electron microscopy analysis was performed as described previously [18].

## Results

### Zebrafish *agr2* Is Exclusively Expressed in Intestinal Goblet Cells

**Figure 1:**
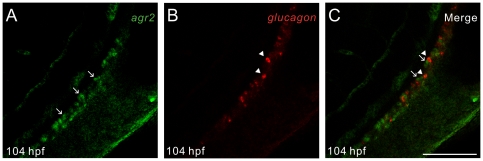
Zebrafish *agr2* is solely expressed in intestinal goblet cells. Fluorescent whole-mount double *in situ* hybridization was conducted on 104-hours post fertilization (hpf) embryos using *agr2* (green) and *glucagon* (red) as RNA probes. Confocal images were recorded using excitation/emission wavelengths of 494/517 nm for fluorescein (A) and 550/570 nm for cyanine 3 (B). Merged image is shown (C). Arrows indicate *agr2*-expressing goblet cells and arrowheads specify *glucagon*-expressing enteroendocrine cells. Scale bars represent 100 µm.

Mouse AGR2 is expressed in MSI1-positive intestinal stem cells and in all three intestinal secretory cell lineages including goblet, Paneth and enteroendocrine cells [11]. There are no Paneth cells in the intestines of zebrafish. We performed fluorescent whole-mount double *in situ* hybridization of zebrafish using *agr2* and *glucagon* as probes. As shown in Figure 1, there is no overlapping expression between *agr2*-expressing goblet cells and *glucagon*-expressing enteroendocrine cells. This result indicates that in contrast to the expression of mouse AGR2 in all intestinal secretory cell lineages, zebrafish *agr2* is solely expressed in intestinal goblet cells.

### Morpholino Antisense Oligomer (MO)-Mediated Knockdown of *agr2* Expression

**Figure 2:**
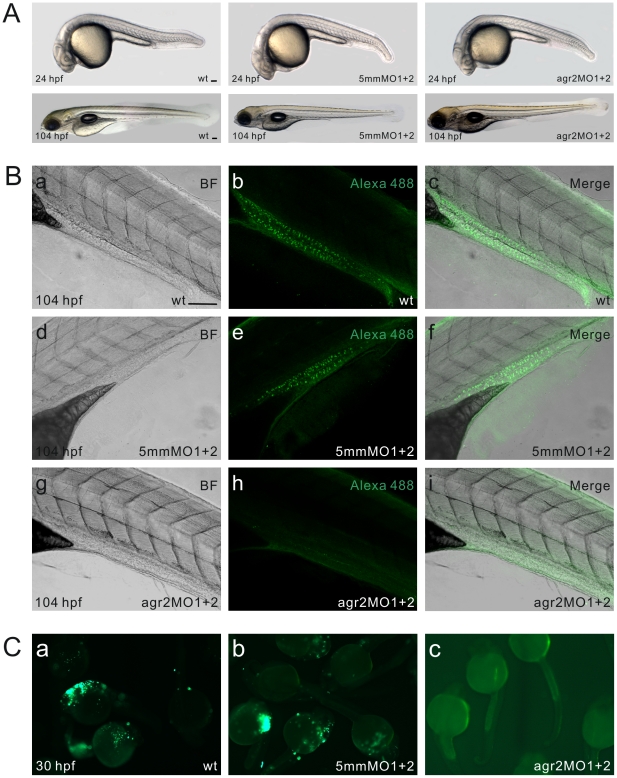
*agr2* morpholino antisense oligomer knockdown analyses. (A) Phenotype comparison among wild type, agr2–5 mmMO1 and 5 mmMO2-coinjected, and agr2-MO1 and agr2-MO2-coinjected embryos at 24 and 104 hpf. (B) Whole-mount immunohistochemistry demonstrates that coinjection of agr2-MO1 and agr2-MO2 prevents the synthesis of Agr2 protein in intestinal goblet cells. Confocal images of either wild type, agr2–5 mmMO1 and 5 mmMO2-coinjected, or agr2-MO1 and agr2-MO2-coinjected 104 hpf embryos were recorded under transmitted mode (a, d, g) or using 494/517 nm excitation/emission wavelengths (b, e, h). Merged images are shown (c, f, i). (C) Green fluorescence was not detected in agr2-MO1, agr2-MO2 and *CMV*-*agr2*-*mo*-*GFP* coinjected (c) 30 hpf embryos, whereas bright green fluorescence was observed in *CMV*-*agr2*-*mo*-*GFP*-injected (a) and agr2–5 mmMO1, agr2–5 mmMO2 and *CMV*-*agr2*-*mo*-*GFP* coinjected (b) 30 hpf embryos. Scale bars represent 100 µm.

To investigate *agr2* function, two agr2-specific translational MOs (agr2-MO1 and agr2-MO2) were designed to inhibit its protein synthesis. Five mismatches were introduced in these two agr2-MOs and used as controls in this study. Initial experiments performed to determine the optimal required doses demonstrated that injection of 7.8 ng of agr2-MO1 (*n* = 61) or agr2-MO2 (*n* = 116) was required to achieve a high morphant rate (88.5% and 81.0%, respectively) as determined by Alcian blue staining (data not shown). However, coinjection of 3.9 ng each of agr2-MO1 and agr2-MO2 can obtain a similarly high (87.0%, *n* = 132) morphant rate. These results demonstrate that there is a synergistic effect between the two translational agr2-MOs. Therefore, we coinjected 3.9 ng each of agr2-MO1 and agr2-MO2 to generate *agr2* morphants in the following experiments. Global morphology of *agr2* morphants was similar to that of embryos that had been coinjected with agr2–5 mmMO1 and 5 mmMO2 and un-injected wild-type embryos at 24 or 104 hours post fertilization (hpf) (Figure 2A). To test the effectiveness of agr2-MOs in preventing the expression of *agr2*, we conducted whole-mount immunohistochemistry using antiserum against salmon Agr2 [21]. Agr2-expressing goblet cells were detected at 104 hpf in the mid-intestines of wild type embryos and in embryos that had been coinjected with both agr2–5 mmMO1 and 5 mmMO2 (Figure 2B). No Agr2-expressing goblet cells were detected in intestines of 104 hpf *agr2* morphants. To further demonstrate the specificity of the agr2-MOs, we fused the *agr2*-MO sequence in front of the *GFP* start codon. We detected no green fluorescence in 30-hpf embryos (*n* = 72) that had been coinjected with 3.9 ng each of agr2-MO1 and agr2-MO2 and 100 pg of *CMV-agr2*-*mo*-*GFP* expression plasmid (Figure 2C). By contrast, bright GFP fluorescence was detected in 78% embryos (*n* = 59) that had been coinjected with the same amount of the agr2–5 mmMO1 and agr2–5 mmMO2 and *CMV*-*agr2*-*mo*-*GFP* expression plasmid as well as in 59% embryos (*n* = 58) that had been injected with *CMV*-*agr2*-*mo*-*GFP* expression plasmid alone. These results demonstrated the specificity of the two translational agr2-MOs used in this study.

### Defects in the Maturation of Goblet Cells Were Detected in the Intestines of *agr2* Morphants

**Figure 3:**
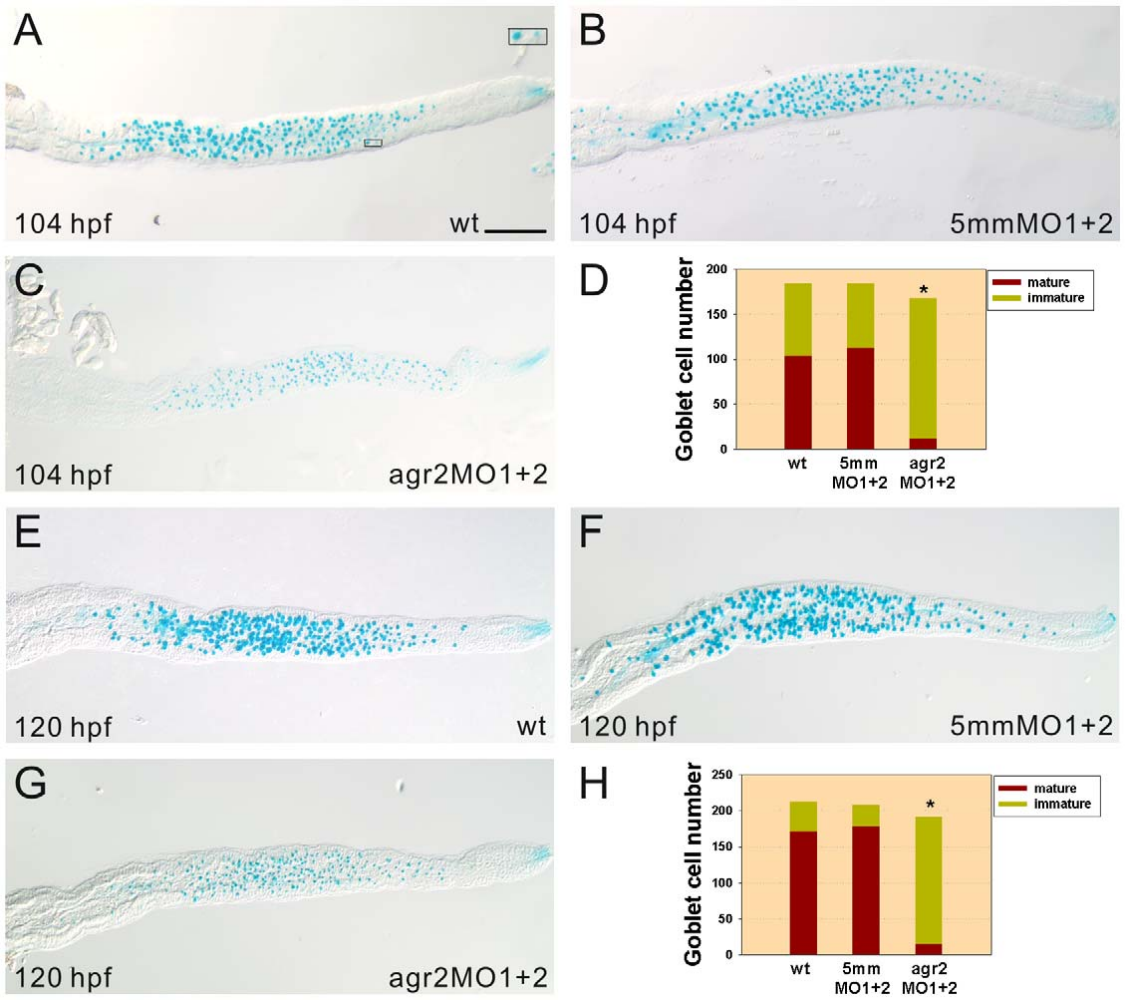
Terminal differentiation of intestinal goblet cells is affected in *agr2* morphants. Significant increases in immature Alcian blue-stained goblet cell numbers were detected in 104- (C, *n* = 46) and 120-hpf (G, *n* = 23) *agr2* morphants compared to those in either 104- (A, *n* = 40) and 120-hpf (E, *n* = 30) wild type or in 104- (B, *n* = 41) and 120-hpf (F, *n* = 27) agr2–5 mmMO1 and 5 mmMO2-coinjected embryos. Inset shows a mature and an immature goblet cell. Comparison of both immature and mature goblet cell numbers among *agr2* morphants, wild type or agr2–5 mmMO1 and 5 mmMO2-coinjected embryos at 104 and 120 hpf are shown (D, H). A Student's *t*-test was conducted to compare immature goblet cell numbers in *agr2* morphants with those in wild type or agr2–5 mmMO1 and 5 mmMO2-coinjected embryos. *p<0.001. Scale bars represent 100 µm.

To investigate the role of *agr2* in the differentiation of goblet cells, we used Alcian blue staining to detect sulfated and carboxylated sialomucins in intestinal goblet cells. Differentiation of goblet cells involves the initial formation of pre-goblet cells containing few mucous granules that later become mature goblet cells which possess abundant mucous granules. We defined Alcian blue-stained goblet cells with an area equal to or larger than 29.22±1.41 µm^2^ (*n* = 10) as mature goblet cells and those with an area less than this value as immature goblet cells. Approximately 93% of the goblet cells were immature in intestines of 104-hpf *agr2* morphants (*n* = 46), compared to 41% (*n* = 41) and 46% (*n* = 40) in intestines of agr2–5 mmMO1 and 5 mmMO2-coinjected and wild type embryos, respectively (Figure 3A–D). A similarly high percentage (92%, *n* = 23) of immature goblet cells remained in intestines of 120-hpf *agr2* morphants compared to 15% (*n* = 27) and 21% (*n* = 30) in intestines of agr2–5 mmMO1 and 5 mmMO2-coinjected and wild type embryos, respectively (Figure 3E–H). However, total intestinal goblet cell number, including both mature and immature goblet cell numbers, was similar among 104- and 120-hpf *agr2* morphants, agr2–5 mmMO1 and 5 mmMO2-coinjected embryos, and wild type embryos.

**Figure 4:**
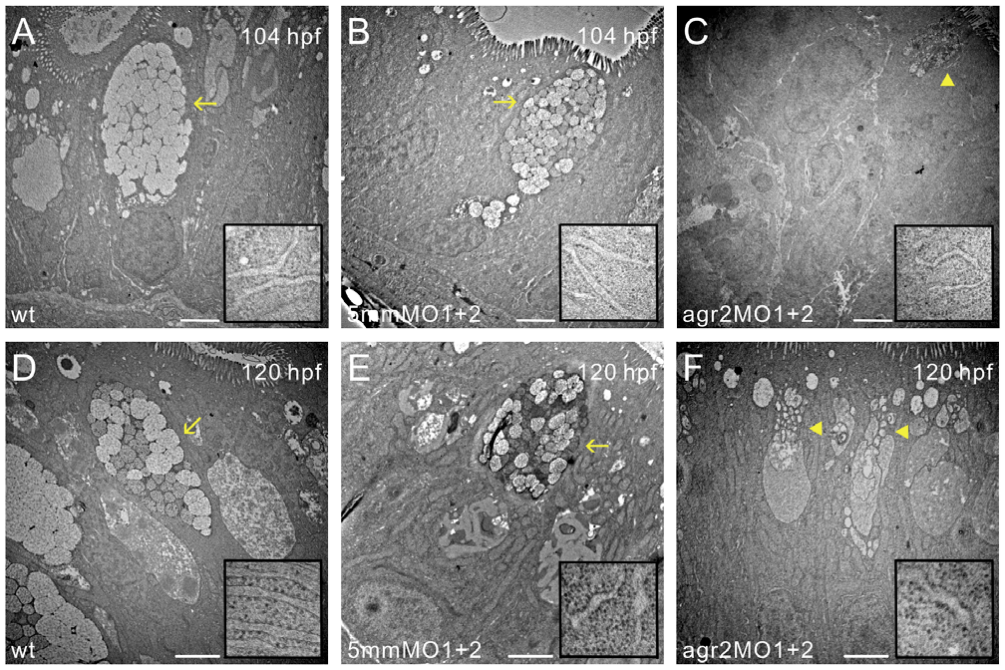
Transmission electron microscopy shows abnormal goblet cell structures in *agr2* morphants. Mid-intestinal images of 104- and 120-hpf wild type (A, D), agr2–5 mmMO1 and 5 mmMO2-coinjected (B, E), and *agr2* morphants (C, F) are shown. ER ultrastructure at higher magnification is shown in insets. Arrows indicate mature goblet cells and arrowheads denote immature goblet cells. Scale bars represent 2 µm.

We used transmission electron microscopy to better define goblet cell ultrastructure in the mid-intestines of 104- and 120-hpf *agr2* morphants, agr2–5 mmMO1 and 5 mmMO2-coinjected embryos and wild type embryos (Figure 4). In the mid-intestines of 104- and 120-hpf wild type and agr2–5 mmMO1 and 5 mmMO2-coinjected embryos, mature goblet cells with many mucous granules that group into swollen thecae can be detected, while in the mid-intestines of 104- and 120-hpf *agr2* morphants, the majority of goblet cells were pre-goblet cells with a small number of mucous granules.

**Figure 5:**
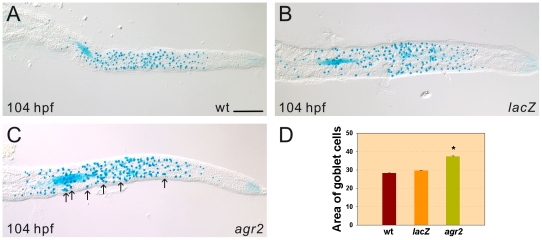
Enlarged areas of mature Alcian-blue stained goblet cells are detected in *agr2*-overexpressed embryos. Substantially increased areas of mature Alcian blue-stained intestinal goblet cells in 42% 104-hpf *agr2*-overexpressed (C, *n* = 29) embryos compared to *lacZ*-overexpressed (B, *n* = 20) embryos and wild type (A, *n* = 18) embryos were observed. Comparison of the area of Alcian blue-stained goblet cells in wild type, *agr2*- and *lacZ*-overexpressed embryos is shown (D). Arrows indicate examples of Alcian-blue stained goblet cells with enlarged areas. Student's *t*-test was conducted and *p<0.001. Scale bars represent 100 µm.

We also conducted an *agr2* overexpression study to investigate its effects on terminal differentiation of goblet cells. Alcian blue-stained mature goblet cells with enlarged areas were detected in intestines of 42% 104-hpf *agr2*-overexpressed (37.22±0.47 µm^2^, *n* = 29) embryos as compared to that in respective *lacZ*-overexpressed (29.64±0.16 µm^2^, *n* = 20) embryos or wild type (28.30±0.15 µm^2^, *n* = 18) embryos (Figure 5).

Taken together, these results demonstrate that *agr2* expression is essential for terminal differentiation of goblet cells in the intestines of zebrafish embryos and overexpression of *agr2* promotes mucin production.

### Knockdown of *agr2* Expression and *agr2* Overexpression Do Not Induce Expression of Unfolded Protein Response (UPR) Pathway Members

**Figure 6:**
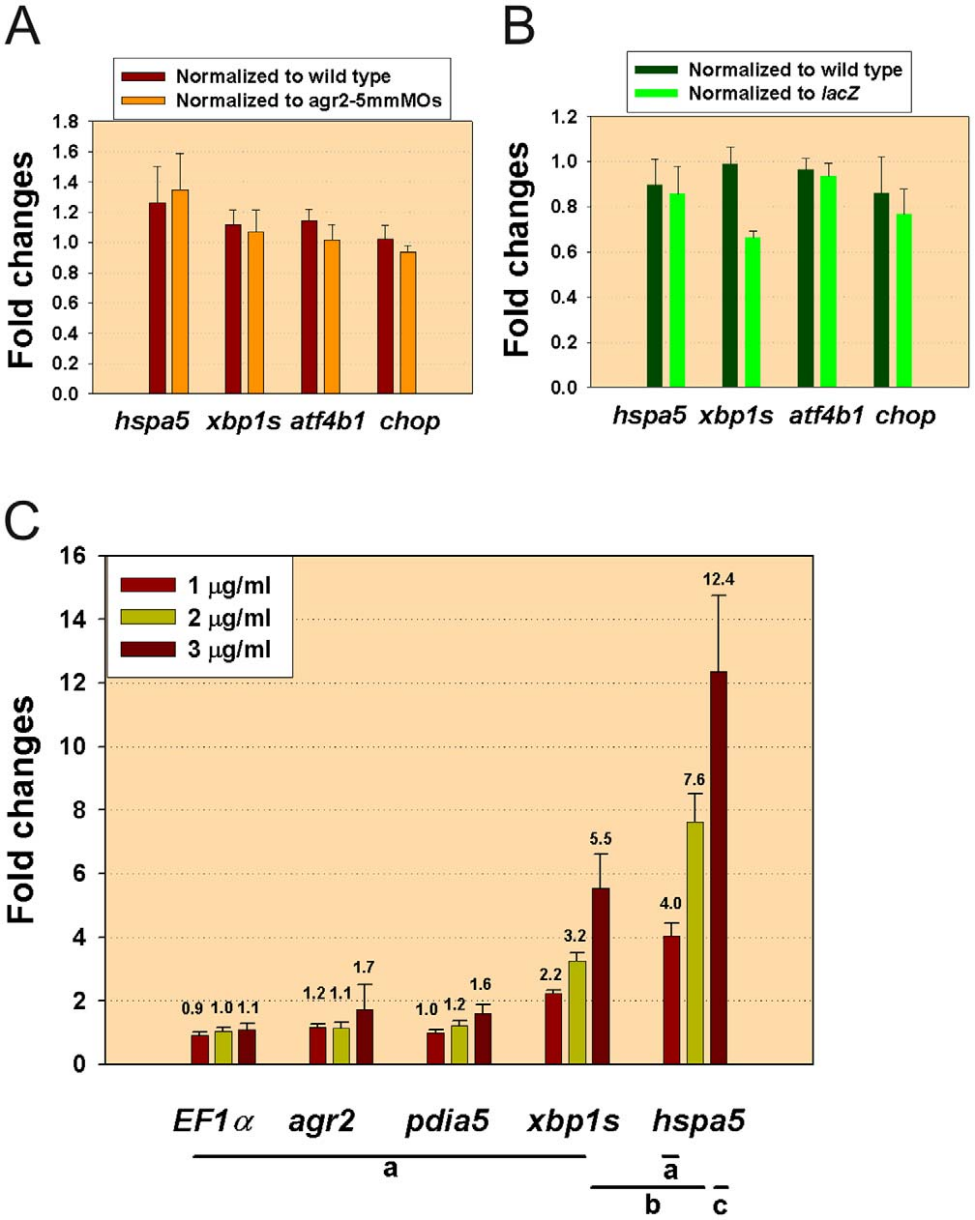
*agr2* expression is not induced by tunicamycin treatment and perturbation of *agr2* level does not induce ER stress. (A) Expression level of members of the UPR pathway (*hspa5*, *xbp1s*, *atf4b1*, *chop*) was not altered in *agr2* morphants when normalized to wild type or to agr2–5 mmMO1 and 5 mmMO2-coinjected 104-hpf embryos by qPCR. (B) Expression of members of the UPR pathway (*hspa5*, *xbp1s*, *atf4b1*, *chop*) was not altered in *agr2*-overexpressed 104-hpf embryos when normalized to wild type or to *lacZ*-overexpressed embryos by qPCR. (C) *agr2* expression level was not significantly induced upon 3 µg/ml tunicamycin treatment by qPCR. One-way analysis of variance and Tukey's honestly significant different method (T-method) were conducted among different genes after treatment with different concentrations of tunicamycin [27]. Different letters (a, b, c) indicate a statistically significant difference (*p*<0.05). Average gene expression in fold changes for respective gene is indicated. Error bars indicate the standard error.

Previous studies had different results regarding induction of ER stress in *Agr2^−/−^* mice, so we investigated whether perturbing *agr2* expression will induce ER stress in zebrafish embryos [12], [13]. To cope with ER stress, genes involved in the UPR pathway are induced. We investigated the mRNA expression levels of members of the UPR pathway, including the *hspa5* gene, which encodes ER chaperone GRP78; c/enhancer binding protein homologous protein (*chop*); activating transcription factor 4b1 (*atf4b1*); and the spliced form of X box-binding protein 1 (*xbp1s*). Mildly increased expression levels (ranging from 1.1–1.3 fold) of *hspa5*, *xbp1s* and *atf4b1* were identified in *agr2* morphants when normalized to wild type or to agr2–5 mmMO1 and 5 mmMO2-coinjected embryos (Figure 6A and Information S1). However, no alteration in *chop* expression level was detected, and the observed mild increases in gene expression levels of *hspa5*, *xbp1s*, and *atfb1* did not reach statistical significance after a Student's *t*-test comparison. In addition, normal ER ultrastructure was observed in goblet cells of 104- and 120-hpf *agr2* morphants (Insets in Figure 4). Similarly, no induction of expression of *hspa5*, *xbp1s*, *atf4b1*, and *chop* could be detected in 104-hpf *agr2*-overexpressed embryos when normalized to wild type or to *lacZ*-overexpressed embryos (Figure 6B and Information S2). These results demonstrate that neither *agr2* deficiency nor *agr2* overexpression can induce ER stress and UPR in zebrafish embryos.

We also treated embryos with different concentration (1–3 µg/ml) of tunicamycin, which inhibits ER protein glycosylation and induces ER stress. We measured respective expression level of *xbp1s*, *hspa5*, *agr2*, and protein disulfide isomerase family A, member 5 (*pdia5*), which is an ER quality control system component gene and *EF1α* control gene in 120-hpf embryos that had been respectively treated with different concentration of tunicamycin (Figure 6C and Information S3). Mildly increased expression levels (ranging from 1.6–1.7 fold) of *agr2* and *pdia5* were identified in 3 µg/ml tunicamycin treated 120-hpf embryos, however the observed mild increases in gene expression levels did not reach statistical significance after one-way analysis of variance using Tukey's honestly significant different method. While significantly increased expression levels (ranging from 5.5–12.4 fold) of *xbp1s* and *hspa5* were detected in 120-hpf embryos that had been treated with the same concentration of tunicamycin. These results indicate that *agr2* expression was not significantly induced in response to ER stress.

### Agr2 Is Not Required for Intestinal Cell Proliferation

**Figure 7:**
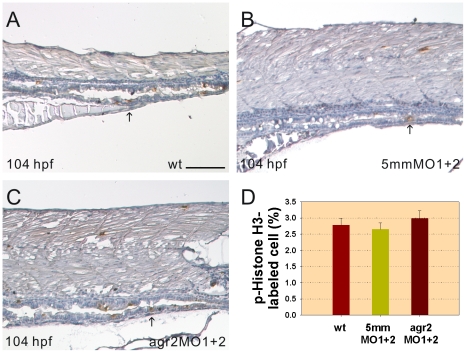
Intestinal cell proliferation is not altered in *agr2* morphants. Images of p-Histone H3-stained cells in the mid-intestines and posterior intestines of 104-hpf wild type embryos (A), agr2–5 mmMO1 and 5 mmMO2-coinjected embryos (B) and *agr2* morphants (C) are shown. Arrows indicate p-Histone H3-stained cells. (D) Comparison of the percentages of p-Histone H3-stained M phase cells among wild type embryos, agr2–5 mmMO1 and 5 mmMO2-coinjected embryos, and *agr2* morphants is shown. Error bars indicate the standard error. Scale bars represent 100 µm.

Activated *AGR2* expression was identified in different human cancer cells and human AGR2 was shown to promote cell growth [5], [6], [7], so we performed p-Histone H3 immunostaining to investigate the role of *agr2* in zebrafish intestinal cell proliferation (Figure 7). A similar percentage of p-Histone H3-stained M phase cells were detected in the mid- and posterior intestines of 104-hpf *agr2* morphants (2.98%, *n* = 5), agr2–5 mmMO1 and 5 mmMO2-coinjected embryos (2.65%, *n* = 6) and wild type embryos (2.78%, *n* = 5). These results indicate that Agr2 is not required for intestinal cell proliferation in zebrafish embryos.

## Discussion

In this study, we characterized the role of zebrafish Agr2 in intestinal cell proliferation and differentiation. Our loss-of-function and overexpression studies indicate that Agr2 is required for the terminal differentiation of goblet cells in zebrafish embryonic intestines.

### Zebrafish Agr2 Is Required for Terminal Differentiation of Intestinal Goblet Cells

Although zebrafish Agr2 shared high (72–80%) amino acid sequence similarities with those of human and mouse AGR2 [17], its distribution in intestinal cell types is different than that of mouse AGR2. A previous study showed that mouse AGR2 is expressed in MSI1-positive intestinal stem cells and all intestinal secretory cell lineages including goblet, Paneth and enteroendocrine cells [11]. In contrast to mammalian intestines, zebrafish intestines only contain 2 secretory cell types, goblet and enteroendocrine cells, and 1 absorptive cell type, enterocytes [16]. Zebrafish intestines do not possess crypts of Lieberkuhn or Paneth cells. Fluorescent whole-mount double *in situ* hybridization further confirmed our previous finding that in zebrafish embryonic intestines, *agr2* is exclusively expressed in goblet cells.

Using *agr2*-specific morpholino antisense oligomers to knockdown *agr2* expression, we demonstrated that Agr2 is required for the maturation but not for the initial differentiation of intestinal goblet cells. Over 90% of goblet cells in both 104- and 120-hpf *agr2* morphants are immature Alcian-blue stained goblet cells, while the total goblet cell number was the same in *agr2* morphants and control embryos (Figure 3). Ultrastructural analyses on goblet cells in the mid-intestines by transmission electron microscope (TEM) further demonstrated the existence of immature pre-goblet cells containing few mucous granules in both 104- and 120-hpf *agr2* morphants (Figure 4). In addition, a significant 1.3-fold increase in areas of mature Alcian-blue stained goblet cells was identified in intestines of 104-hpf *agr2*-overexpressed embryos, suggesting that *agr2* overexpression promotes mucin production (Figure 5). Overall, our results are consistent with a previous study showing that mouse AGR2 is essential for the production of mucin and intestinal mucus [13]. Owing to the lack of a suitable antibody which could recognize Muc2 in intestines of zebrafish embryos, we were not able to confirm changes in Muc2 protein level when *agr2* expression was perturbed in zebrafish embryos.

Mouse AGR2 localizes to the lumen of ER and is a member of the protein disulfide isomerase family, which is involved in mucin processing. Therefore, we investigated the role of zebrafish Agr2 in ER stress [13]. In contrast to previous studies in two different cell lines, *agr2* expression was mildly (1.7 fold) induced upon 3 µg/ml tunicamycin treatment in zebrafish embryos (Figure 6C) [12], [23]. In addition, similar induction level (1.6 fold) of *pdia5* gene which is involved in ER quality control/folding machinery was detected in 3 µg/ml tunicamycin treated zebrafish embryos (Figure 6C). These results indicate that in contrast to *xbp1s* and *hspa5*, expression levels of PDI members such as *agr2* and *pdia5* were mildly induced, which did not reach statistical significant level in response to ER stress.

In *agr2* knockdown embryos, we detected mildly (1.1 to 1.3-fold) increased expression levels in several members of the UPR pathway, including *hspa5*, *xbp1s* and *atf4b1*. These expression level increases did not reach statistical significance after a Student's *t*-test comparison (Figure 6A). This result was in agreement with previous reports showing that mild increases in expression of Bip and *XBP1s* were observed in *Agr2* siRNA knockdown SU86.86 and HEK-293T cells [12], [23]. Our results also support a previous study showing statistically insignificant increases in the expression levels of *Hspa5*, *Ero1*, *Atf4*, and *Atf6* in *Agr2^−/−^* mice [13]. We also detected no alteration in expression levels of genes involved in ER degradation of misfolded proteins (ERAD) including ER degradation-enhancing α-mannosidase-like lectins 1 (*EDEM1*), *calnexin* and *calreticulin* in either *agr2* knockdown or *agr2* overexpressed embryos, these results are different from a previous report conducted in HEK-293T cells (data not shown) [23]. In contrast to the existence of ER stress-induced rough ER dilation in the intestinal goblet cells of *Winnie* or *Eeyore* mutant mice harboring a *Muc2* missense mutation [24], our TEM results showed normal ER ultrastructure in *agr2* morphants (Figure 4). Overall, our results suggest that Agr2 may not play important roles in the control of ER homeostasis in zebrafish embryos.

Both ulcerative colitis and Crohn's disease, two forms of chronic human inflammatory bowel disease (IBD), were shown to contain reduced mucosal thickness and low numbers of goblet cells [25]. *Muc2* knockout mice spontaneously developed a mild colitis and treatment with cytotoxic luminal agents like DSS led to severe colitis [26]. Similarly, *Agr2*-deficient mice were highly susceptible to DSS-induced colitis due to abnormalities in MUC2 synthesis [13]. In the future, it will be interesting to investigate if DSS treatment can promote development of intestinal inflammation in *agr2* knockdown zebrafish embryos and the possibility of using *agr2* deficient zebrafish as a model to study IBD.

Results of p-Histone H3 immunohistochemistry demonstrated that Agr2 plays no role in regulating intestinal cell proliferation in zebrafish embryos (Figure 7). Our finding is consistent with a previous study showing that no decreased proliferation in either the small intestine or the colon could be detected in germline *Agr2^−/−^* mice [12]. However, these results are inconsistent with studies in cancer cell lines showing that human AGR2 can promote cell growth by modulating expression of *cyclin D1* and components of the EGF and HIPPO signaling pathways [6], [7]. This discrepancy may be due to the abnormal status of cancer cells versus normal intestinal cells.

In conclusion, our loss- and gain-of-function studies demonstrate that zebrafish Agr2 functions like its mouse homolog in regulating terminal differentiation of intestinal goblet cells. However, Agr2 may not play important roles in the control of ER homeostasis in zebrafish embryos.

## Supporting Information

Information S1 **Raw qPCR data regarding expression levels of members of the UPR pathway in wild type embryos or embryos that had been injected with either agr2–5 mmMO1 and 5 mmMO2 or agr2 MO1 and MO2.** Crossing point (Cp) values of respective *atf4b1*, *chop*, *xbp1s*, *hspa5*, and *β-actin* detected in either 104-hpf wild type, agr2–5 mmMO1 and 5 mmMO2-coinjected, or agr2 MO1 and MO2-coinjected embryos are shown. NTC represents no template control.
(DOC)

Information S2 **Raw qPCR data regarding expression levels of members of the UPR pathway in embryos that had been respectively injected with either** ***lacZ*** **or** ***agr2*** **mRNA.** Crossing point (Cp) values of respective *atf4b1*, *chop*, *xbp1s*, *hspa5*, and *β-actin* detected in 104-hpf embryos that had been injected with either 250 pg *lacZ* or 200/250 pg *agr2* mRNA are shown. NTC represents no template control.
(DOC)

Information S3 **Raw qPCR data regarding expression levels of** ***agr2*** **,** ***ef1α*** **and members of the UPR pathway in untreated wild type or tunicamycin-treated embryos.** Crossing point (Cp) values of respective *ef1α*, *agr2*, *pdia5*, *xbp1s*, *hspa5*, and *β-actin* detected in 120-hpf untreated wild type or embryos that had been treated with 1–3 µg/ml tunicamycin for 24 h are shown. NTC represents no template control. Result of one-way analysis of variance and Tukey's honestly significant deferent method (T-method) is also shown.
(DOC)
