# Supplementary material for: Zebrafish Agr2 Is Required for Terminal Differentiation of Intestinal Goblet Cells
Source: PLoS One. 2012 Apr 13;7(4):e34408. doi: 10.1371/journal.pone.0034408 (PMC3326001; doi:10.1371/journal.pone.0034408)
Supplement: Information S1 — Raw qPCR data regarding expression levels of members of the UPR pathway in wild type embryos or embryos that had been injected with either agr2–5 mmMO1 and 5 mmMO2 or agr2 MO1 and MO2. Crossing point (Cp) values of respective atf4b1, chop, xbp1s, hspa5, and β-actin detected in either 104-hpf wild type, agr2–5 mmMO1 and 5 mmMO2-coinjected, or agr2 MO1 and MO2-coinjected embryos are shown. NTC represents no template control. (DOC) [file pone.0034408.s001.doc]

Crossing point (Cp) values of ATF4b1 and b-actin after zebrafish embryos injected with 5mm MO1+5mmMO2 or 2 MOs

| Experiment 1 | | | | Experiment 2 | | | | Experiment 3 | | | |
| --- | --- | --- | --- | --- | --- | --- | --- | --- | --- | --- | --- |
| Name | Cp | Standard | Status | Name | Cp | Standard | Status | Name | Cp | Standard | Status |
| wt-ATF4 | 27.19 | 0 |  | wt-ATF4 | 27.52 | 0 |  | wt-ATF4 | 25.63 | 0 |  |
| wt-ATF4 | 26.74 | 0 |  | wt-ATF4 | 27.48 | 0 |  | wt-ATF4 | 25.54 | 0 |  |
| wt-ATF4 | 27.57 | 0 |  | wt-ATF4 | 27.34 | 0 |  | wt-ATF4 | 25.59 | 0 |  |
| 5mm-ATF4 | 27.51 | 0 |  | 5mm-ATF4 | 27.63 | 0 |  | 5mm-ATF4 | 25.7 | 0 |  |
| 5mm-ATF4 | 27.78 | 0 |  | 5mm-ATF4 | 27.65 | 0 |  | 5mm-ATF4 | 25.84 | 0 |  |
| 5mm-ATF4 | 27.75 | 0 |  | 5mm-ATF4 | 28 | 0 |  | 5mm-ATF4 | 25.7 | 0 |  |
| MO-ATF4 | 26.7 | 0 |  | MO-ATF4 | 27.01 | 0 |  | MO-ATF4 | 25.75 | 0 |  |
| MO-ATF4 | 26.64 | 0 |  | MO-ATF4 | 27.12 | 0 |  | MO-ATF4 | 25.7 | 0 |  |
| MO-ATF4 | 26.99 | 0 |  | MO-ATF4 | 27.27 | 0 |  | MO-ATF4 | 25.67 | 0 |  |
| wt-b-act | 19.65 | 0 |  | wt-b-act | 20.68 | 0 |  | wt-b-act | 17.59 | 0 |  |
| wt-b-act | 20 | 0 |  | wt-b-act | 20.67 | 0 |  | wt-b-act | 18.06 | 0 |  |
| wt-b-act | 20.5 | 0 |  | wt-b-act | 20.71 | 0 |  | wt-b-act | 17.76 | 0 |  |
| 5mm-b-act | 21.51 | 0 |  | 5mm-b-act | 21 | 0 |  | 5mm-b-act | 18.08 | 0 |  |
| 5mm-b-act | 21.3 | 0 |  | 5mm-b-act | 20.89 | 0 |  | 5mm-b-act | 18.14 | 0 |  |
| 5mm-b-act | 21.15 | 0 |  | 5mm-b-act | 20.94 | 0 |  | 5mm-b-act | 18.17 | 0 |  |
| MO-b-act | 20.05 | 0 |  | MO-b-act | 20.79 | 0 |  | MO-b-act | 18.15 | 0 |  |
| MO-b-act | 20.25 | 0 |  | MO-b-act | 20.85 | 0 |  | MO-b-act | 18.14 | 0 |  |
| MO-b-act | 19.95 | 0 |  | MO-b-act | 20.75 | 0 |  | MO-b-act | 18.17 | 0 |  |
| ATF4 NTC |  | 0 |  | ATF4 NTC |  | 0 |  | ATF4 NTC |  | 0 |  |
| b-act NTC |  | 0 |  | b-act NTC |  | 0 |  | b-act NTC |  | 0 |  |

| Experiment 4 | | | | Experiment 5 | | | | Experiment 6 | | | |
| --- | --- | --- | --- | --- | --- | --- | --- | --- | --- | --- | --- |
| Name | Cp | Standard | Status | Name | Cp | Standard | Status | Name | Cp | Standard | Status |
| wt-ATF4 | 25.78 | 0 |  | wt-ATF4 | 23.01 | 0 |  | wt-ATF4 | 22.93 | 0 |  |
| wt-ATF4 | 25.74 | 0 |  | wt-ATF4 | 23.11 | 0 |  | wt-ATF4 | 23.07 | 0 |  |
| wt-ATF4 | 25.83 | 0 |  | wt-ATF4 | 23.07 | 0 |  | wt-ATF4 | 23.02 | 0 |  |
| 5mm-ATF4 | 25.64 | 0 |  | 5mm-ATF4 | 22.59 | 0 |  | 5mm-ATF4 | 23.61 | 0 |  |
| 5mm-ATF4 | 25.49 | 0 |  | 5mm-ATF4 | 22.79 | 0 |  | 5mm-ATF4 | 23.65 | 0 |  |
| 5mm-ATF4 | 25.54 | 0 |  | 5mm-ATF4 | 22.7 | 0 |  | 5mm-ATF4 | 23.61 | 0 |  |
| MO-ATF4 | 25.89 | 0 |  | MO-ATF4 | 23.52 | 0 |  | MO-ATF4 | 23.19 | 0 |  |
| MO-ATF4 | 25.94 | 0 |  | MO-ATF4 | 23.6 | 0 |  | MO-ATF4 | 23.33 | 0 |  |
| MO-ATF4 | 25.99 | 0 |  | MO-ATF4 | 23.58 | 0 |  | MO-ATF4 | 23.2 | 0 |  |
| wt-b-act | 18.07 | 0 |  | wt-b-act | 17.83 | 0 |  | wt-b-act | 17.79 | 0 |  |
| wt-b-act | 18.02 | 0 |  | wt-b-act | 17.88 | 0 |  | wt-b-act | 17.77 | 0 |  |
| wt-b-act | 18.01 | 0 |  | wt-b-act | 17.82 | 0 |  | wt-b-act | 17.91 | 0 |  |
| 5mm-b-act | 17.94 | 0 |  | 5mm-b-act | 17.65 | 0 |  | 5mm-b-act | 18.21 | 0 |  |
| 5mm-b-act | 18.31 | 0 |  | 5mm-b-act | 17.5 | 0 |  | 5mm-b-act | 18.47 | 0 |  |
| 5mm-b-act | 18.13 | 0 |  | 5mm-b-act | 17.6 | 0 |  | 5mm-b-act | 18.26 | 0 |  |
| MO-b-act | 18.03 | 0 |  | MO-b-act | 18.3 | 0 |  | MO-b-act | 17.91 | 0 |  |
| MO-b-act | 18 | 0 |  | MO-b-act | 18.29 | 0 |  | MO-b-act | 18.08 | 0 |  |
| MO-b-act | 18.04 | 0 |  | MO-b-act | 18.23 | 0 |  | MO-b-act | 18.11 | 0 |  |
| ATF4 NTC |  | 0 |  | ATF4-NTC |  | 0 |  | ATF4-NTC |  | 0 |  |
| b-act NTC |  | 0 |  | b-act-NTC |  | 0 |  | b-act-NTC |  | 0 |  |

Crossing point (Cp) values of CHOP and b-actin after zebrafish embryos injected with 5mm MO1+5mmMO2 or 2 MOs

| Experiment 1 | | | | Experiment 2 | | | | Experiment 3 | | | |
| --- | --- | --- | --- | --- | --- | --- | --- | --- | --- | --- | --- |
| Name | Cp | Standard | Status | Name | Cp | Standard | Status | Name | Cp | Standard | Status |
| wt-CHOP | 36.22 | 0 |  | wt-CHOP | 28.27 | 0 |  | wt-CHOP | 28.86 | 0 |  |
| wt-CHOP | 36.65 | 0 |  | wt-CHOP | 28.42 | 0 |  | wt-CHOP | 28.95 | 0 |  |
| wt-CHOP | 36.61 | 0 |  | wt-CHOP | 28.25 | 0 |  | wt-CHOP | 28.64 | 0 |  |
| 5mm-CHOP | 36.66 | 0 |  | 5mm-CHOP | 28.71 | 0 |  | 5mm-CHOP | 28.25 | 0 |  |
| 5mm-CHOP | 36.59 | 0 |  | 5mm-CHOP | 28.69 | 0 |  | 5mm-CHOP | 28.28 | 0 |  |
| 5mm-CHOP | 36.52 | 0 |  | 5mm-CHOP | 28.64 | 0 |  | 5mm-CHOP | 28.56 | 0 |  |
| MO-CHOP | 36.48 | 0 |  | MO-CHOP | 28.57 | 0 |  | MO-CHOP | 28.17 | 0 |  |
| MO-CHOP | 36.69 | 0 |  | MO-CHOP | 28.63 | 0 |  | MO-CHOP | 28.29 | 0 |  |
| MO-CHOP | 36.58 | 0 |  | MO-CHOP | 28.61 | 0 |  | MO-CHOP | 28.24 | 0 |  |
| wt-b-act | 17.59 | 0 |  | wt-b-act | 17.79 | 0 |  | wt-b-act | 18.3 | 0 |  |
| wt-b-act | 18.06 | 0 |  | wt-b-act | 17.77 | 0 |  | wt-b-act | 18.29 | 0 |  |
| wt-b-act | 17.76 | 0 |  | wt-b-act | 17.91 | 0 |  | wt-b-act | 18.23 | 0 |  |
| 5mm-b-act | 18.08 | 0 |  | 5mm-b-act | 18.21 | 0 |  | 5mm-b-act | 17.83 | 0 |  |
| 5mm-b-act | 18.14 | 0 |  | 5mm-b-act | 18.47 | 0 |  | 5mm-b-act | 17.88 | 0 |  |
| 5mm-b-act | 18.17 | 0 |  | 5mm-b-act | 18.26 | 0 |  | 5mm-b-act | 17.82 | 0 |  |
| MO-b-act | 18.15 | 0 |  | MO-b-act | 17.91 | 0 |  | MO-b-act | 17.65 | 0 |  |
| MO-b-act | 18.14 | 0 |  | MO-b-act | 18.08 | 0 |  | MO-b-act | 17.5 | 0 |  |
| MO-b-act | 18.17 | 0 |  | MO-b-act | 18.11 | 0 |  | MO-b-act | 17.6 | 0 |  |
| b-act NTC |  | 0 |  | b-act-NTC |  | 0 |  | b-act-NTC |  | 0 |  |
| CHOP NTC |  | 0 |  | CHOP-NTC |  | 0 |  | CHOP-NTC |  | 0 |  |

Crossing point (Cp) values of xbp1-s and b-actin after zebrafish embryos injected with 5mm MO1+5mmMO2 or 2 MOs

| Experiment 1 | | | | Experiment 2 | | | | Experiment 3 | | | | Experiment 4 | | | |
| --- | --- | --- | --- | --- | --- | --- | --- | --- | --- | --- | --- | --- | --- | --- | --- |
| Name | Cp | Standard | Status | Name | Cp | Standard | Status | Name | Cp | Standard | Status | Name | Cp | Standard | Status |
| wt-xbp1-s | 26.57 | 0 |  | wt-xbp1-s | 25.55 | 0 |  | wt-xbp1-s | 26.09 | 0 |  | wt-xbp1-s | 26.73 | 0 |  |
| wt-xbp1-s | 26.79 | 0 |  | wt-xbp1-s | 25.54 | 0 |  | wt-xbp1-s | 26.1 | 0 |  | wt-xbp1-s | 26.89 | 0 |  |
| wt-xbp1-s | 26.75 | 0 |  | wt-xbp1-s | 25.69 | 0 |  | wt-xbp1-s | 26.19 | 0 |  | wt-xbp1-s | 26.82 | 0 |  |
| 5mm-xbp1-s | 26.21 | 0 |  | 5mm-xbp1-s | 26.16 | 0 |  | 5mm-xbp1-s | 25.74 | 0 |  | 5mm-xbp1-s | 27.13 | 0 |  |
| 5mm-xbp1-s | 26.42 | 0 |  | 5mm-xbp1-s | 26.05 | 0 |  | 5mm-xbp1-s | 25.7 | 0 |  | 5mm-xbp1-s | 27.3 | 0 |  |
| 5mm-xbp1-s | 26.3 | 0 |  | 5mm-xbp1-s | 26.13 | 0 |  | 5mm-xbp1-s | 25.7 | 0 |  | 5mm-xbp1-s | 27.22 | 0 |  |
| MO-xbp1-s | 26.71 | 0 |  | MO-xbp1-s | 25.73 | 0 |  | MO-xbp1-s | 25.01 | 0 |  | MO-xbp1-s | 27.03 | 0 |  |
| MO-xbp1-s | 26.69 | 0 |  | MO-xbp1-s | 25.83 | 0 |  | MO-xbp1-s | 24.88 | 0 |  | MO-xbp1-s | 27.1 | 0 |  |
| MO-xbp1-s | 26.64 | 0 |  | MO-xbp1-s | 25.96 | 0 |  | MO-xbp1-s | 24.97 | 0 |  | MO-xbp1-s | 26.99 | 0 |  |
| wt-b-act | 18.07 | 0 |  | wt-b-act | 17.79 | 0 |  | wt-b-act | 18.3 | 0 |  | wt-b-act | 17.59 | 0 |  |
| wt-b-act | 18.02 | 0 |  | wt-b-act | 17.77 | 0 |  | wt-b-act | 18.29 | 0 |  | wt-b-act | 18.06 | 0 |  |
| wt-b-act | 18.01 | 0 |  | wt-b-act | 17.91 | 0 |  | wt-b-act | 18.23 | 0 |  | wt-b-act | 17.76 | 0 |  |
| 5mm-b-act | 17.94 | 0 |  | 5mm-b-act | 18.21 | 0 |  | 5mm-b-act | 17.83 | 0 |  | 5mm-b-act | 18.08 | 0 |  |
| 5mm-b-act | 18.31 | 0 |  | 5mm-b-act | 18.47 | 0 |  | 5mm-b-act | 17.88 | 0 |  | 5mm-b-act | 18.14 | 0 |  |
| 5mm-b-act | 18.13 | 0 |  | 5mm-b-act | 18.26 | 0 |  | 5mm-b-act | 17.82 | 0 |  | 5mm-b-act | 18.17 | 0 |  |
| MO-b-act | 18.03 | 0 |  | MO-b-act | 17.91 | 0 |  | MO-b-act | 17.65 | 0 |  | MO-b-act | 18.15 | 0 |  |
| MO-b-act | 18 | 0 |  | MO-b-act | 18.08 | 0 |  | MO-b-act | 17.5 | 0 |  | MO-b-act | 18.14 | 0 |  |
| MO-b-act | 18.04 | 0 |  | MO-b-act | 18.11 | 0 |  | MO-b-act | 17.6 | 0 |  | MO-b-act | 18.17 | 0 |  |
| xbp1-s NTC |  | 0 |  | xbp1-s-NTC |  | 0 |  | xbp1-s-NTC |  | 0 |  | xbp1-s-NTC |  | 0 |  |
| b-act NTC |  | 0 |  | b-act-NTC |  | 0 |  | b-act-NTC |  | 0 |  | b-act-NTC |  | 0 |  |

Crossing point (Cp) values of HSPA5 and b-actin after zebrafish embryos injected with 5mm MO1+5mmMO2 or 2 MOs

| Experiment 1 | | | | Experiment 2 | | | |
| --- | --- | --- | --- | --- | --- | --- | --- |
| Name | Cp | Standard | Status | Name | Cp | Standard | Status |
| wt-HSPA5 | 23.53 | 0 |  | wt-HSPA5 | 24.01 | 0 |  |
| wt-HSPA5 | 23.48 | 0 |  | wt-HSPA5 | 24.08 | 0 |  |
| wt-HSPA5 | 23.49 | 0 |  | wt-HSPA5 | 24.07 | 0 |  |
| 5mm-HSPA5 | 24.08 | 0 |  | 5mm-HSPA5 | 23.73 | 0 |  |
| 5mm-HSPA5 | 24.08 | 0 |  | 5mm-HSPA5 | 23.7 | 0 |  |
| 5mm-HSPA5 | 24.15 | 0 |  | 5mm-HSPA5 | 23.68 | 0 |  |
| MO-HSPA5 | 23.65 | 0 |  | MO-HSPA5 | 22.82 | 0 |  |
| MO-HSPA5 | 23.74 | 0 |  | MO-HSPA5 | 22.76 | 0 |  |
| MO-HSPA5 | 23.64 | 0 |  | MO-HSPA5 | 22.75 | 0 |  |
| wt-b-act | 17.79 | 0 |  | wt-b-act | 18.3 | 0 |  |
| wt-b-act | 17.77 | 0 |  | wt-b-act | 18.29 | 0 |  |
| wt-b-act | 17.91 | 0 |  | wt-b-act | 18.23 | 0 |  |
| 5mm-b-act | 18.21 | 0 |  | 5mm-b-act | 17.88 | 0 |  |
| 5mm-b-act | 18.47 | 0 |  | 5mm-b-act | 17.82 | 0 |  |
| 5mm-b-act | 18.26 | 0 |  | 5mm-b-act | 17.83 | 0 |  |
| MO-b-act | 17.91 | 0 |  | MO-b-act | 17.65 | 0 |  |
| MO-b-act | 18.08 | 0 |  | MO-b-act | 17.5 | 0 |  |
| MO-b-act | 18.11 | 0 |  | MO-b-act | 17.6 | 0 |  |
| b-act-NTC |  | 0 |  | b-act-NTC |  | 0 |  |
| HSPA5-NTC |  | 0 |  | HSPA5-NTC |  | 0 |  |
