# Supplementary material for: Zebrafish Agr2 Is Required for Terminal Differentiation of Intestinal Goblet Cells
Source: PLoS One. 2012 Apr 13;7(4):e34408. doi: 10.1371/journal.pone.0034408 (PMC3326001; doi:10.1371/journal.pone.0034408)
Supplement: Information S2 — Raw qPCR data regarding expression levels of members of the UPR pathway in embryos that had been respectively injected with either lacZ or agr2 mRNA. Crossing point (Cp) values of respective atf4b1, chop, xbp1s, hspa5, and β-actin detected in 104-hpf embryos that had been injected with either 250 pg lacZ or 200/250 pg agr2 mRNA are shown. NTC represents no template control. (DOC) [file pone.0034408.s002.doc]

Crossing point (Cp) values of ATF4b1 and b-actin after zebrafish embryos injected with 250pg lacZ or 250pg or 200pg agr2 mRNA

| Experiment 1 | | | | Experiment 2 | | | |
| --- | --- | --- | --- | --- | --- | --- | --- |
| Name | Cp | Standard | Status | Name | Cp | Standard | Status |
| wt-ATF4 | 25.74 | 0 |  | wt-ATF4 | 26.42 | 0 |  |
| wt-ATF4 | 25.69 | 0 |  | wt-ATF4 | 26.24 | 0 |  |
| 250pg lacZ-ATF4 | 25.84 | 0 |  | 250pg lacZ-ATF4 | 26.42 | 0 |  |
| 250pg lacZ-ATF4 | 25.88 | 0 |  | 250pg lacZ-ATF4 | 26.29 | 0 |  |
| 250pg agr2-ATF4 | 25.88 | 0 |  | 250pg agr2-ATF4 | 25.71 | 0 |  |
| 250pg agr2-ATF4 | 25.81 | 0 |  | 250pg agr2-ATF4 | 25.74 | 0 |  |
| 200pg agr2-ATF4 | 25.69 | 0 |  | 200pg agr2-ATF4 | 25.84 | 0 |  |
| 200pg agr2-ATF4 | 25.71 | 0 |  | 200pg agr2-ATF4 | 25.86 | 0 |  |
| wt-b-act | 20.88 | 0 |  | wt-b-act | 21.21 | 0 |  |
| wt-b-act | 20.84 | 0 |  | wt-b-act | 21.28 | 0 |  |
| 250pg lacZ-b-act | 21.04 | 0 |  | 250pg lacZ-b-act | 21.31 | 0 |  |
| 250pg lacZ-b-act | 21.1 | 0 |  | 250pg lacZ-b-act | 21.28 | 0 |  |
| 250pg agr2-b-act | 20.76 | 0 |  | 250pg agr2-b-act | 20.79 | 0 |  |
| 250pg agr2-b-act | 20.72 | 0 |  | 250pg agr2-b-act | 20.73 | 0 |  |
| 200pg agr2-b-act | 20.71 | 0 |  | 200pg agr2-b-act | 20.77 | 0 |  |
| 200pg agr2-b-act | 20.74 | 0 |  | 200pg agr2-b-act | 20.77 | 0 |  |
| b-act NTC |  | 0 |  | b-act NTC |  | 0 |  |
| ATF4 NTC |  | 0 |  | ATF4 NTC |  | 0 |  |

Crossing point (Cp) values of CHOP and b-actin after zebrafish embryos injected with 250pg lacZ or 250pg or 200pg agr2 mRNA

| Experiment 1 | | | | Experiment 2 | | | |
| --- | --- | --- | --- | --- | --- | --- | --- |
| Name | Cp | Standard | Status | Name | Cp | Standard | Status |
| wt-1-CHOP | 30.01 | 0 |  | wt-1-CHOP | 30.42 | 0 |  |
| wt-1-CHOP | 30.02 | 0 |  | wt-1-CHOP | 30.19 | 0 |  |
| 250pg lacZ-1-CHOP | 30.02 | 0 |  | 250pg lacZ-1-CHOP | 30.51 | 0 |  |
| 250pg lacZ-1-CHOP | 29.92 | 0 |  | 250pg lacZ-1-CHOP | 30.15 | 0 |  |
| 250pg agr2-CHOP | 30.14 | 0 |  | 250pg agr2-CHOP | 30.56 | 0 |  |
| 250pg agr2-CHOP | 29.9 | 0 |  | 250pg agr2-CHOP | 30.57 | 0 |  |
| 200pg agr2-CHOP | 29.44 | 0 |  | 200pg agr2-CHOP | 30.61 | 0 |  |
| 200pg agr2-CHOP | 29.59 | 0 |  | 200pg agr2-CHOP | 30.32 | 0 |  |
| wt-1-b-act | 20.88 | 0 |  | wt-1-b-act | 21.21 | 0 |  |
| wt-1-b-act | 20.84 | 0 |  | wt-1-b-act | 21.28 | 0 |  |
| 250pg lacZ-1-b-act | 21.04 | 0 |  | 250pg lacZ-1-b-act | 21.31 | 0 |  |
| 250pg lacZ-1-b-act | 21.1 | 0 |  | 250pg lacZ-1-b-act | 21.28 | 0 |  |
| 250pg agr2-b-act | 20.76 | 0 |  | 250pg agr2-b-act | 20.79 | 0 |  |
| 250pg agr2-b-act | 20.72 | 0 |  | 250pg agr2-b-act | 20.73 | 0 |  |
| 200pg agr2-b-act | 20.71 | 0 |  | 200pg agr2-b-act | 20.77 | 0 |  |
| 200pg agr2-b-act | 20.74 | 0 |  | 200pg agr2-b-act | 20.77 | 0 |  |
| b-act NTC |  | 0 |  | b-act NTC |  | 0 |  |
| CHOP NTC |  | 0 |  | CHOP NTC |  | 0 |  |

Crossing point (Cp) values of HSPA5 and b-actin after zebrafish embryos injected with 250pg lacZ or 250pg or 200pg agr2 mRNA

| Name | Cp | Standard | Status | Name | Cp | Standard | Status | Name | Cp | Standard | Status |
| --- | --- | --- | --- | --- | --- | --- | --- | --- | --- | --- | --- |
| wt-HSPA5 | 22.21 | 0 |  | wt-HSPA5 | 24.51 | 0 |  | wt-HSPA5 | 25.52 | 0 |  |
| wt-HSPA5 | 22.24 | 0 |  | wt-HSPA5 | 24.6 | 0 |  | wt-HSPA5 | 25.57 | 0 |  |
| wt-HSPA5 | 22.19 | 0 |  | 250pg lacZ-HSPA5 | 25.04 | 0 |  | 250pg lacZ-HSPA5 | 25.58 | 0 |  |
| 250pg lacZ- HSPA5 | 22.47 | 0 |  | 250pg lacZ-HSPA5 | 24.97 | 0 |  | 250pg lacZ-HSPA5 | 25.5 | 0 |  |
| 250pg lacZ- HSPA5 | 22.43 | 0 |  | 250pg agr2-HSPA5 | 24.56 | 0 |  | 250pg agr2-HSPA5 | 24.6 | 0 |  |
| 250pg lacZ- HSPA5 | 22.44 | 0 |  | 250pg agr2-HSPA5 | 24.57 | 0 |  | 250pg agr2-HSPA5 | 24.6 | 0 |  |
| 250pg agr2- HSPA5 | 22.01 | 0 |  | 200pg agr2-HSPA5 | 24.72 | 0 |  | 200pg agr2-HSPA5 | 24.61 | 0 |  |
| 250pg agr2- HSPA5 | 22.02 | 0 |  | 200pg agr2-HSPA5 | 24.65 | 0 |  | 200pg agr2-HSPA5 | 24.69 | 0 |  |
| 250pg agr2- HSPA5 | 21.98 | 0 |  | wt-b-act | 20.88 | 0 |  | wt-b-act | 21.21 | 0 |  |
| 200pg agr2-1-HSPA5 | 22.49 | 0 |  | wt-b-act | 20.84 | 0 |  | wt-b-act | 21.28 | 0 |  |
| 200pg agr2-1-HSPA5 | 22.48 | 0 |  | 250pg lacZ-b-act | 21.04 | 0 |  | 250pg lacZ-b-act | 21.31 | 0 |  |
| 200pg agr2-1-HSPA5 | 22.51 | 0 |  | 250pg lacZ-b-act | 21.1 | 0 |  | 250pg lacZ-b-act | 21.28 | 0 |  |
| 200pg agr2-2-HSPA5 | 22.77 | 0 |  | 250pg agr2-b-act | 20.71 | 0 |  | 250pg agr2-b-act | 20.77 | 0 |  |
| 200pg agr2-2-HSPA5 | 22.56 | 0 |  | 250pg agr2-b-act | 20.74 | 0 |  | 250pg agr2-b-act | 20.77 | 0 |  |
| 200pg agr2-2-HSPA5 | 22.79 | 0 |  | 200pg agr2-b-act | 20.76 | 0 |  | 200pg agr2-b-act | 20.79 | 0 |  |
| 200pg agr2-3-HSPA5 | 22.61 | 0 |  | 200pg agr2-b-act | 20.72 | 0 |  | 200pg agr2-b-act | 20.73 | 0 |  |
| 200pg agr2-3-HSPA5 | 22.59 | 0 |  | b-act NTC |  | 0 |  | b-act NTC |  | 0 |  |
| 200pg agr2-3-HSPA5 | 22.63 | 0 |  | HSPA5 NTC |  | 0 |  | HSPA5 NTC |  | 0 |  |
| wt-b-act | 17.97 | 0 |  |  |  |  |  |  |  |  |  |
| wt-b-act | 17.98 | 0 |  |  |  |  |  |  |  |  |  |
| wt-b-act | 17.94 | 0 |  |  |  |  |  |  |  |  |  |
| 250pg lacZ-b-act | 18.5 | 0 |  |  |  |  |  |  |  |  |  |
| 250pg lacZ-b-act | 18.47 | 0 |  |  |  |  |  |  |  |  |  |
| 250pg lacZ-b-act | 18.43 | 0 |  |  |  |  |  |  |  |  |  |
| 250pg agr2-b-act | 17.68 | 0 |  |  |  |  |  |  |  |  |  |
| 250pg agr2-b-act | 17.65 | 0 |  |  |  |  |  |  |  |  |  |
| 250pg agr2-b-act | 17.62 | 0 |  |  |  |  |  |  |  |  |  |
| 200pg agr2-1-b-act | 17.61 | 0 |  |  |  |  |  |  |  |  |  |
| 200pg agr2-1-b-act | 17.63 | 0 |  |  |  |  |  |  |  |  |  |
| 200pg agr2-1-b-act | 17.59 | 0 |  |  |  |  |  |  |  |  |  |
| 200pg agr2-2-b-act | 17.73 | 0 |  |  |  |  |  |  |  |  |  |
| 200pg agr2-2-b-act | 17.74 | 0 |  |  |  |  |  |  |  |  |  |
| 200pg agr2-2-b-act | 17.73 | 0 |  |  |  |  |  |  |  |  |  |
| 200pg agr2-3-b-act | 17.49 | 0 |  |  |  |  |  |  |  |  |  |
| 200pg agr2-3-b-act | 17.46 | 0 |  |  |  |  |  |  |  |  |  |
| 200pg agr2-3-b-act | 17.46 | 0 |  |  |  |  |  |  |  |  |  |
| b-act-NTC |  | 0 |  |  |  |  |  |  |  |  |  |
| HSPA5-NTC |  | 0 |  |  |  |  |  |  |  |  |  |

Crossing point (Cp) values of xbp1-s and b-actin after zebrafish embryos injected with 250pg lacZ or 250pg or 200pg agr2 mRNA

| Experiment 1 | | | | Experiment 2 | | | | Experiment 3 | | | | Experiment 4 | | | |
| --- | --- | --- | --- | --- | --- | --- | --- | --- | --- | --- | --- | --- | --- | --- | --- |
| Name | Cp | Standard | Status | Name | Cp | Standard | Status | Name | Cp | Standard | Status | Name | Cp | Standard | Status |
| wt-xbp1-s | 25.86 | 0 |  | wt-xbp1-s | 25.6 | 0 |  | wt-xbp1-s | 26.86 | 0 |  | wt-xbp1-s | 27.51 | 0 |  |
| wt-xbp1-s | 25.9 | 0 |  | wt-xbp1-s | 25.64 | 0 |  | wt-xbp1-s | 26.86 | 0 |  | wt-xbp1-s | 27.44 | 0 |  |
| wt-xbp1-s | 25.84 | 0 |  | wt-xbp1-s | 25.66 | 0 |  | 250pg lacZ-xbp1-s | 26.93 | 0 |  | 250pg lacZ-xbp1-s | 27.5 | 0 |  |
| 250pg lacZ-xbp1-s | 24.95 | 0 |  | 250pg lacZ-xbp1-s | 25.46 | 0 |  | 250pg lacZ-xbp1-s | 26.88 | 0 |  | 250pg lacZ-xbp1-s | 27.65 | 0 |  |
| 250pg lacZ-xbp1-s | 24.97 | 0 |  | 250pg lacZ-xbp1-s | 25.42 | 0 |  | 250pg agr2-xbp1-s | 27.72 | 0 |  | 250pg agr2-xbp1-s | 27.31 | 0 |  |
| 250pg lacZ-xbp1-s | 25.11 | 0 |  | 250pg lacZ-xbp1-s | 25.53 | 0 |  | 250pg agr2-xbp1-s | 27.72 | 0 |  | 250pg agr2-xbp1-s | 27.27 | 0 |  |
| 250pg agr2-xbp1-s | 24.78 | 0 |  | 250pg agr2-xbp1-s | 24.99 | 0 |  | 200pg agr2-xbp1-s | 27.42 | 0 |  | 200pg agr2-xbp1-s | 27.6 | 0 |  |
| 250pg agr2-xbp1-s | 24.92 | 0 |  | 250pg agr2-xbp1-s | 24.96 | 0 |  | 200pg agr2-xbp1-s | 27.43 | 0 |  | 200pg agr2-xbp1-s | 27.48 | 0 |  |
| 250pg agr2-xbp1-s | 25.04 | 0 |  | 250pg agr2-xbp1-s | 25 | 0 |  | wt-b-act | 20.88 | 0 |  | wt-b-act | 21.21 | 0 |  |
| 200pg agr2-1-xbp1-s | 25.03 | 0 |  | 200pg agr2-1-xbp1-s | 25.03 | 0 |  | wt-b-act | 20.84 | 0 |  | wt-b-act | 21.28 | 0 |  |
| 200pg agr2-1-xbp1-s | 25.04 | 0 |  | 200pg agr2-1-xbp1-s | 25.04 | 0 |  | 250pg lacZ-b-act | 21.04 | 0 |  | 250pg lacZ-b-act | 21.31 | 0 |  |
| 200pg agr2-1-xbp1-s | 25.03 | 0 |  | 200pg agr2-1-xbp1-s | 25.03 | 0 |  | 250pg lacZ-b-act | 21.1 | 0 |  | 250pg lacZ-b-act | 21.28 | 0 |  |
| 200pg agr2-2-xbp1-s | 25.26 | 0 |  | 200pg agr2-2-xbp1-s | 25.26 | 0 |  | 250pg agr2-b-act | 20.76 | 0 |  | 250pg agr2-b-act | 20.79 | 0 |  |
| 200pg agr2-2-xbp1-s | 25.12 | 0 |  | 200pg agr2-2-xbp1-s | 25.12 | 0 |  | 250pg agr2-b-act | 20.72 | 0 |  | 250pg agr2-b-act | 20.73 | 0 |  |
| 200pg agr2-2-xbp1-s | 25.24 | 0 |  | 200pg agr2-2-xbp1-s | 25.24 | 0 |  | 200pg agr2-b-act | 20.71 | 0 |  | 200pg agr2-b-act | 20.77 | 0 |  |
| 200pg agr2-3-xbp1-s | 25.01 | 0 |  | 200pg agr2-3-xbp1-s | 25.01 | 0 |  | 200pg agr2-b-act | 20.74 | 0 |  | 200pg agr2-b-act | 20.77 | 0 |  |
| 200pg agr2-3-xbp1-s | 24.85 | 0 |  | 200pg agr2-3-xbp1-s | 24.85 | 0 |  | xbp1-s NTC |  | 0 |  | xbp1-s NTC |  | 0 |  |
| 200pg agr2-3-xbp1-s | 24.94 | 0 |  | 200pg agr2-3-xbp1-s | 24.94 | 0 |  | b-act NTC |  | 0 |  | b-act NTC |  | 0 |  |
| wt-b-act | 18.43 | 0 |  | wt-b-act | 17.97 | 0 |  |  |  |  |  |  |  |  |  |
| wt-b-act | 18.3 | 0 |  | wt-b-act | 17.98 | 0 |  |  |  |  |  |  |  |  |  |
| wt-b-act | 18.3 | 0 |  | wt-b-act | 17.94 | 0 |  |  |  |  |  |  |  |  |  |
| 250pg lacZ-b-act | 18.25 | 0 |  | 250pg lacZ-b-act | 18.5 | 0 |  |  |  |  |  |  |  |  |  |
| 250pg lacZ-b-act | 18.17 | 0 |  | 250pg lacZ-b-act | 18.47 | 0 |  |  |  |  |  |  |  |  |  |
| 250pg lacZ-b-act | 18.22 | 0 |  | 250pg lacZ-b-act | 18.43 | 0 |  |  |  |  |  |  |  |  |  |
| 250pg agr2-b-act | 17.63 | 0 |  | 250pg agr2-b-act | 17.68 | 0 |  |  |  |  |  |  |  |  |  |
| 250pg agr2-b-act | 17.61 | 0 |  | 250pg agr2-b-act | 17.65 | 0 |  |  |  |  |  |  |  |  |  |
| 250pg agr2-b-act | 17.56 | 0 |  | 250pg agr2-b-act | 17.62 | 0 |  |  |  |  |  |  |  |  |  |
| 200pg agr2-1-b-act | 17.61 | 0 |  | 200pg agr2-1-b-act | 17.61 | 0 |  |  |  |  |  |  |  |  |  |
| 200pg agr2-1-b-act | 17.63 | 0 |  | 200pg agr2-1-b-act | 17.63 | 0 |  |  |  |  |  |  |  |  |  |
| 200pg agr2-1-b-act | 17.59 | 0 |  | 200pg agr2-1-b-act | 17.59 | 0 |  |  |  |  |  |  |  |  |  |
| 200pg agr2-2-b-act | 17.73 | 0 |  | 200pg agr2-2-b-act | 17.73 | 0 |  |  |  |  |  |  |  |  |  |
| 200pg agr2-2-b-act | 17.74 | 0 |  | 200pg agr2-2-b-act | 17.74 | 0 |  |  |  |  |  |  |  |  |  |
| 200pg agr2-2-b-act | 17.73 | 0 |  | 200pg agr2-2-b-act | 17.73 | 0 |  |  |  |  |  |  |  |  |  |
| 200pg agr2-3-b-act | 17.49 | 0 |  | 200pg agr2-3-b-act | 17.49 | 0 |  |  |  |  |  |  |  |  |  |
| 200pg agr2-3-b-act | 17.46 | 0 |  | 200pg agr2-3-b-act | 17.46 | 0 |  |  |  |  |  |  |  |  |  |
| 200pg agr2-3-b-act | 17.46 | 0 |  | 200pg agr2-3-b-act | 17.46 | 0 |  |  |  |  |  |  |  |  |  |
| xbp1-s NTC |  | 0 |  | xbp1-s NTC |  | 0 |  |  |  |  |  |  |  |  |  |
| b-act-NTC |  | 0 |  | b-act-NTC |  | 0 |  |  |  |  |  |  |  |  |  |
