# Supplementary material for: Zebrafish Agr2 Is Required for Terminal Differentiation of Intestinal Goblet Cells
Source: PLoS One. 2012 Apr 13;7(4):e34408. doi: 10.1371/journal.pone.0034408 (PMC3326001; doi:10.1371/journal.pone.0034408)
Supplement: Information S3 — Raw qPCR data regarding expression levels of agr2 , ef1α and members of the UPR pathway in untreated wild type or tunicamycin-treated embryos. Crossing point (Cp) values of respective ef1α, agr2, pdia5, xbp1s, hspa5, and β-actin detected in 120-hpf untreated wild type or embryos that had been treated with 1–3 µg/ml tunicamycin for 24 h are shown. NTC represents no template control. Result of one-way analysis of variance and Tukey's honestly significant deferent method (T-method) is also shown. (DOC) [file pone.0034408.s003.doc]

Crossing point (Cp) values of agr2 and b-actin after zebrafish embryos treated with 1-3 μg/ml tunicamycin or 5 μg/ml DMSO

| Experiment 1 | | | | Experiment 2 | | | | Experiment 3 | | | |
| --- | --- | --- | --- | --- | --- | --- | --- | --- | --- | --- | --- |
| Name | Cp | Standard | Status | Name | Cp | Standard | Status | Name | Cp | Standard | Status |
| agr2-1μg | 23.8 | 0 |  | agr2-1μg | 24.55 | 0 |  | agr2-1μg | 23.14 | 0 |  |
| agr2-1μg | 23.95 | 0 |  | agr2-1μg | 24.64 | 0 |  | agr2-1μg | 23.28 | 0 |  |
| agr2-1μg | 23.98 | 0 |  | agr2-2μg | 24.34 | 0 |  | agr2-2μg | 23.22 | 0 |  |
| agr2-2μg | 24.16 | 0 |  | agr2-2μg | 24.34 | 0 |  | agr2-2μg | 23.2 | 0 |  |
| agr2-2μg | 24.48 | 0 |  | agr2-3μg | 23.15 | 0 |  | agr2-3μg | 23.96 | 0 |  |
| agr2-2μg | 24.28 | 0 |  | agr2-3μg | 23.12 | 0 |  | agr2-3μg | 24.04 | 0 |  |
| agr2-3μg | 23.92 | 0 |  | agr2-DMSO | 24.28 | 0 |  | agr2-DMSO | 24.03 | 0 |  |
| agr2-3μg | 23.91 | 0 |  | agr2-DMSO | 24.46 | 0 |  | agr2-DMSO | 24.13 | 0 |  |
| agr2-3μg | 23.96 | 0 |  | b act-1μg | 21.72 | 0 |  | b act-1μg | 20.17 | 0 |  |
| agr2-DMSO | 24.51 | 0 |  | b act-1μg | 21.75 | 0 |  | b act-1μg | 20.54 | 0 |  |
| agr2-DMSO | 24.47 | 0 |  | b act-2μg | 21.19 | 0 |  | b act-2μg | 20.47 | 0 |  |
| agr2-DMSO | 24.49 | 0 |  | b act-2μg | 21.21 | 0 |  | b act-2μg | 20.5 | 0 |  |
| b act-1μg | 20.79 | 0 |  | b act-3μg | 19.87 | 0 |  | b act-3μg | 23.45 | 0 |  |
| b act-1μg | 20.98 | 0 |  | b act-3μg | 20.03 | 0 |  | b act-3μg | 23.53 | 0 |  |
| b act-1μg | 20.86 | 0 |  | b act-DMSO | 21.44 | 0 |  | b act-DMSO | 20.96 | 0 |  |
| b act-2μg | 21.54 | 0 |  | b act-DMSO | 21.52 | 0 |  | b act-DMSO | 21.17 | 0 |  |
| b act-2μg | 21.35 | 0 |  | b act-NTC |  | 0 |  | b act-NTC |  | 0 |  |
| b act-2μg | 21.29 | 0 |  | agr2-NTC |  | 0 |  | agr2-NTC | 37.63 | 0 | > - Late Cp call (last five cycles) has higher uncertainty |
| b act-3μg | 20.43 | 0 |  |  |  |  |  |  |  |  |  |
| b act-3μg | 20.44 | 0 |  |  |  |  |  |  |  |  |  |
| b act-3μg | 20.26 | 0 |  |  |  |  |  |  |  |  |  |
| b act-DMSO | 20.9 | 0 |  |  |  |  |  |  |  |  |  |
| b act-DMSO | 20.88 | 0 |  |  |  |  |  |  |  |  |  |
| b act-DMSO | 20.84 | 0 |  |  |  |  |  |  |  |  |  |
| b act-NTC |  | 0 |  |  |  |  |  |  |  |  |  |
| agr2-NTC | 35.96 | 0 | > - Late Cp call (last five cycles) has higher uncertainty |  |  |  |  |  |  |  |  |

| Experiment 4 | | | | Experiment 5 | | | | Experiment 6 | | | |
| --- | --- | --- | --- | --- | --- | --- | --- | --- | --- | --- | --- |
| Name | Cp | Standard | Status | Name | Cp | Standard | Status | Name | Cp | Standard | Status |
| agr2-1μg | 21.51 | 0 |  | agr2-1μg | 22.24 | 0 |  | agr2-1μg | 22.05 | 0 |  |
| agr2-1μg | 21.5 | 0 |  | agr2-1μg | 22.26 | 0 |  | agr2-1μg | 22.03 | 0 |  |
| agr2-2μg | 22.33 | 0 |  | agr2-2μg | 22.1 | 0 |  | agr2-2μg | 22.51 | 0 |  |
| agr2-2μg | 22.19 | 0 |  | agr2-2μg | 21.95 | 0 |  | agr2-2μg | 22.61 | 0 |  |
| agr2-3μg | 22.02 | 0 |  | agr2-3μg | 21.63 | 0 |  | agr2-3μg | 23 | 0 |  |
| agr2-3μg | 22.1 | 0 |  | agr2-3μg | 21.54 | 0 |  | agr2-3μg | 23.06 | 0 |  |
| agr2-DMSO | 21.98 | 0 |  | agr2-DMSO | 21.78 | 0 |  | agr2-DMSO | 22.87 | 0 |  |
| agr2-DMSO | 21.91 | 0 |  | agr2-DMSO | 21.76 | 0 |  | agr2-DMSO | 22.9 | 0 |  |
| b act-1μg | 19.34 | 0 |  | b act-1μg | 18.68 | 0 |  | b act-1μg | 19.07 | 0 |  |
| b act-1μg | 19.25 | 0 |  | b act-1μg | 18.72 | 0 |  | b act-1μg | 19.06 | 0 |  |
| b act-2μg | 19.45 | 0 |  | b act-2μg | 19.61 | 0 |  | b act-2μg | 18.64 | 0 |  |
| b act-2μg | 19.48 | 0 |  | b act-2μg | 19.57 | 0 |  | b act-2μg | 18.73 | 0 |  |
| b act-3μg | 19.34 | 0 |  | b act-3μg | 18.79 | 0 |  | b act-3μg | 19.01 | 0 |  |
| b act-3μg | 19.29 | 0 |  | b act-3μg | 18.84 | 0 |  | b act-3μg | 19.06 | 0 |  |
| b act-DMSO | 19.49 | 0 |  | b act-DMSO | 18.67 | 0 |  | b act-DMSO | 19.53 | 0 |  |
| b act-DMSO | 19.26 | 0 |  | b act-DMSO | 18.59 | 0 |  | b act-DMSO | 19.49 | 0 |  |
| b act-NTC |  | 0 |  | b act-NTC |  | 0 |  | b act-NTC |  | 0 |  |
| agr2-NTC |  | 0 |  | agr2-NTC | 36.78 | 0 | > - Late Cp call (last five cycles) has higher uncertainty | agr2-NTC |  | 0 |  |

Crossing point (Cp) values of pdia5 and b-actin after zebrafish embryos treated with 1-3 μg/ml tunicamycin or 5 μg/ml DMSO

| Experiment 1 | | | | Experiment 2 | | | | Experiment 3 | | | |
| --- | --- | --- | --- | --- | --- | --- | --- | --- | --- | --- | --- |
| Name | Cp | Standard | Status | Name | Cp | Standard | Status | Name | Cp | Standard | Status |
| pdia5-1μg | 24.46 | 0 |  | pdia5-1μg | 27.02 | 0 |  | pdia5-1μg | 25.61 | 0 |  |
| pdia5-1μg | 24.54 | 0 |  | pdia5-1μg | 27.08 | 0 |  | pdia5-1μg | 25.5 | 0 |  |
| pdia5-2μg | 25.21 | 0 |  | pdia5-2μg | 25.8 | 0 |  | pdia5-2μg | 25.19 | 0 |  |
| pdia5-2μg | 26.74 | 0 |  | pdia5-2μg | 25.96 | 0 |  | pdia5-2μg | 25.21 | 0 |  |
| pdia5-3μg | 26 | 0 |  | pdia5-3μg | 24.79 | 0 |  | pdia5-3μg | 28.52 | 0 |  |
| pdia5-3μg | 26.09 | 0 |  | pdia5-3μg | 24.93 | 0 |  | pdia5-3μg | 28.6 | 0 |  |
| pdia5-DMSO | 25.79 | 0 |  | pdia5-DMSO | 26.33 | 0 |  | pdia5-DMSO | 26.23 | 0 |  |
| pdia5-DMSO | 25.91 | 0 |  | pdia5-DMSO | 26.42 | 0 |  | pdia5-DMSO | 26.31 | 0 |  |
| b act-1μg | 18.57 | 0 |  | b act-1μg | 21.72 | 0 |  | b act-1μg | 20.17 | 0 |  |
| b act-1μg | 18.64 | 0 |  | b act-1μg | 21.75 | 0 |  | b act-1μg | 20.54 | 0 |  |
| b act-2μg | 19.02 | 0 |  | b act-2μg | 21.19 | 0 |  | b act-2μg | 20.47 | 0 |  |
| b act-2μg | 20.04 | 0 |  | b act-2μg | 21.21 | 0 |  | b act-2μg | 20.5 | 0 |  |
| b act-3μg | 22.03 | 0 |  | b act-3μg | 19.87 | 0 |  | b act-3μg | 23.45 | 0 |  |
| b act-3μg | 21.78 | 0 |  | b act-3μg | 20.03 | 0 |  | b act-3μg | 23.53 | 0 |  |
| b act-DMSO | 20.47 | 0 |  | b act-DMSO | 21.44 | 0 |  | b act-DMSO | 20.96 | 0 |  |
| b act-DMSO | 20.2 | 0 |  | b act-DMSO | 21.52 | 0 |  | b act-DMSO | 21.17 | 0 |  |
| b act-NTC |  |  |  | b act-NTC |  | 0 |  | b act-NTC |  | 0 |  |
| pdia5-NTC |  |  |  | pdia5-NTC |  | 0 |  | pdia5-NTC |  | 0 |  |

| Experiment 4 | | | | Experiment 5 | | | | Experiment 6 | | | |
| --- | --- | --- | --- | --- | --- | --- | --- | --- | --- | --- | --- |
| Name | Cp | Standard | Status | Name | Cp | Standard | Status | Name | Cp | Standard | Status |
| pdia5-1μg | 25.13 | 0 |  | pdia5-1μg | 24.03 | 0 |  | pdia5-1μg | 24.93 | 0 |  |
| pdia5-1μg | 25.16 | 0 |  | pdia5-1μg | 24.09 | 0 |  | pdia5-1μg | 24.92 | 0 |  |
| pdia5-2μg | 24.94 | 0 |  | pdia5-2μg | 24.92 | 0 |  | pdia5-2μg | 24.05 | 0 |  |
| pdia5-2μg | 25.04 | 0 |  | pdia5-2μg | 24.92 | 0 |  | pdia5-2μg | 24.07 | 0 |  |
| pdia5-3μg | 24.53 | 0 |  | pdia5-3μg | 23.58 | 0 |  | pdia5-3μg | 24.61 | 0 |  |
| pdia5-3μg | 24.58 | 0 |  | pdia5-3μg | 23.59 | 0 |  | pdia5-3μg | 24.63 | 0 |  |
| pdia5-DMSO | 25.62 | 0 |  | pdia5-DMSO | 24.21 | 0 |  | pdia5-DMSO | 25.15 | 0 |  |
| pdia5-DMSO | 25.76 | 0 |  | pdia5-DMSO | 24.23 | 0 |  | pdia5-DMSO | 25.2 | 0 |  |
| b act-1μg | 19.34 | 0 |  | b act-1μg | 18.68 | 0 |  | b act-1μg | 19.07 | 0 |  |
| b act-1μg | 19.25 | 0 |  | b act-1μg | 18.72 | 0 |  | b act-1μg | 19.06 | 0 |  |
| b act-2μg | 19.45 | 0 |  | b act-2μg | 19.61 | 0 |  | b act-2μg | 18.64 | 0 |  |
| b act-2μg | 19.48 | 0 |  | b act-2μg | 19.57 | 0 |  | b act-2μg | 18.73 | 0 |  |
| b act-3μg | 19.34 | 0 |  | b act-3μg | 18.79 | 0 |  | b act-3μg | 19.01 | 0 |  |
| b act-3μg | 19.29 | 0 |  | b act-3μg | 18.84 | 0 |  | b act-3μg | 19.06 | 0 |  |
| b act-DMSO | 19.49 | 0 |  | b act-DMSO | 18.67 | 0 |  | b act-DMSO | 19.53 | 0 |  |
| b act-DMSO | 19.26 | 0 |  | b act-DMSO | 18.59 | 0 |  | b act-DMSO | 19.49 | 0 |  |
| b act-NTC |  | 0 |  | b act-NTC |  | 0 |  | b act-NTC |  | 0 |  |
| pdia5-NTC |  | 0 |  | pdia5-NTC |  | 0 |  | pdia5-NTC |  | 0 |  |

Crossing point (Cp) values of HSPA5 and b-actin after zebrafish embryos treated with 1-3 μg/ml tunicamycin or 5 μg/ml DMSO

| Epxeriment 1 | | | | Epxeriment 2 | | | | Epxeriment 3 | | | |
| --- | --- | --- | --- | --- | --- | --- | --- | --- | --- | --- | --- |
| Name | Cp | Standard | Status | Name | Cp | Standard | Status | Name | Cp | Standard | Status |
| HSPA5-1μg | 23.25 | 0 |  | HSPA5-1μg | 24.18 | 0 |  | HSPA5-1μg | 22.77 | 0 |  |
| HSPA5-1μg | 23.3 | 0 |  | HSPA5-1μg | 24.22 | 0 |  | HSPA5-1μg | 22.73 | 0 |  |
| HSPA5-1μg | 23.29 | 0 |  | HSPA5-2μg | 22.55 | 0 |  | HSPA5-2μg | 22.59 | 0 |  |
| HSPA5-2μg | 22.77 | 0 |  | HSPA5-2μg | 22.6 | 0 |  | HSPA5-2μg | 22.57 | 0 |  |
| HSPA5-2μg | 22.73 | 0 |  | HSPA5-3μg | 21.09 | 0 |  | HSPA5-3μg | 24.86 | 0 |  |
| HSPA5-2μg | 23 | 0 |  | HSPA5-3μg | 20.98 | 0 |  | HSPA5-3μg | 24.93 | 0 |  |
| HSPA5-3μg | 21.88 | 0 |  | HSPA5-DMSO | 25.56 | 0 |  | HSPA5-DMSO | 25.52 | 0 |  |
| HSPA5-3μg | 21.94 | 0 |  | HSPA5-DMSO | 25.58 | 0 |  | HSPA5-DMSO | 25.57 | 0 |  |
| HSPA5-3μg | 21.8 | 0 |  | b act-1μg | 21.72 | 0 |  | b act-1μg | 20.17 | 0 |  |
| HSPA5-DMSO | 25.42 | 0 |  | b act-1μg | 21.75 | 0 |  | b act-1μg | 20.54 | 0 |  |
| HSPA5-DMSO | 25.5 | 0 |  | b act-2μg | 21.19 | 0 |  | b act-2μg | 20.47 | 0 |  |
| HSPA5-DMSO | 25.3 | 0 |  | b act-2μg | 21.21 | 0 |  | b act-2μg | 20.5 | 0 |  |
| b act-1μg | 20.79 | 0 |  | b act-3μg | 19.87 | 0 |  | b act-3μg | 23.45 | 0 |  |
| b act-1μg | 20.98 | 0 |  | b act-3μg | 20.03 | 0 |  | b act-3μg | 23.53 | 0 |  |
| b act-1μg | 20.86 | 0 |  | b act-DMSO | 21.44 | 0 |  | b act-DMSO | 20.96 | 0 |  |
| b act-2μg | 21.54 | 0 |  | b act-DMSO | 21.52 | 0 |  | b act-DMSO | 21.17 | 0 |  |
| b act-2μg | 21.35 | 0 |  | b act-NTC |  | 0 |  | b act-NTC |  | 0 |  |
| b act-2μg | 21.29 | 0 |  | hspa5-NTC |  | 0 |  | hspa5-NTC |  | 0 |  |
| b act-3μg | 20.43 | 0 |  |  |  |  |  |  |  |  |  |
| b act-3μg | 20.44 | 0 |  |  |  |  |  |  |  |  |  |
| b act-3μg | 20.26 | 0 |  |  |  |  |  |  |  |  |  |
| b act-DMSO | 20.9 | 0 |  |  |  |  |  |  |  |  |  |
| b act-DMSO | 20.88 | 0 |  |  |  |  |  |  |  |  |  |
| b act-DMSO | 20.84 | 0 |  |  |  |  |  |  |  |  |  |
| b act-NTC |  | 0 |  |  |  |  |  |  |  |  |  |
| HSPA5-NTC |  | 0 |  |  |  |  |  |  |  |  |  |

| Epxeriment 4 | | | | Epxeriment 5 | | | | Epxeriment 6 | | | |
| --- | --- | --- | --- | --- | --- | --- | --- | --- | --- | --- | --- |
| Name | Cp | Standard | Status | Name | Cp | Standard | Status | Name | Cp | Standard | Status |
| HSPA5-1μg | 21.98 | 0 |  | HSPA5-1μg | 20.42 | 0 |  | HSPA5-1μg | 21.58 | 0 |  |
| HSPA5-1μg | 21.99 | 0 |  | HSPA5-1μg | 20.45 | 0 |  | HSPA5-1μg | 21.66 | 0 |  |
| HSPA5-2μg | 20.83 | 0 |  | HSPA5-2μg | 20.8 | 0 |  | HSPA5-2μg | 19.89 | 0 |  |
| HSPA5-2μg | 20.95 | 0 |  | HSPA5-2μg | 20.79 | 0 |  | HSPA5-2μg | 19.95 | 0 |  |
| HSPA5-3μg | 19.74 | 0 |  | HSPA5-3μg | 19.12 | 0 |  | HSPA5-3μg | 19.34 | 0 |  |
| HSPA5-3μg | 19.85 | 0 |  | HSPA5-3μg | 19.14 | 0 |  | HSPA5-3μg | 19.27 | 0 |  |
| HSPA5-DMSO | 24.43 | 0 |  | HSPA5-DMSO | 22.79 | 0 |  | HSPA5-DMSO | 23.5 | 0 |  |
| HSPA5-DMSO | 24.28 | 0 |  | HSPA5-DMSO | 22.8 | 0 |  | HSPA5-DMSO | 23.56 | 0 |  |
| b act-1μg | 19.34 | 0 |  | b act-1μg | 18.68 | 0 |  | b act-1μg | 19.07 | 0 |  |
| b act-1μg | 19.25 | 0 |  | b act-1μg | 18.72 | 0 |  | b act-1μg | 19.06 | 0 |  |
| b act-2μg | 19.45 | 0 |  | b act-2μg | 19.61 | 0 |  | b act-2μg | 18.64 | 0 |  |
| b act-2μg | 19.48 | 0 |  | b act-2μg | 19.57 | 0 |  | b act-2μg | 18.73 | 0 |  |
| b act-3μg | 19.34 | 0 |  | b act-3μg | 18.79 | 0 |  | b act-3μg | 19.01 | 0 |  |
| b act-3μg | 19.29 | 0 |  | b act-3μg | 18.84 | 0 |  | b act-3μg | 19.06 | 0 |  |
| b act-DMSO | 19.49 | 0 |  | b act-DMSO | 18.67 | 0 |  | b act-DMSO | 19.53 | 0 |  |
| b act-DMSO | 19.26 | 0 |  | b act-DMSO | 18.59 | 0 |  | b act-DMSO | 19.49 | 0 |  |
| b act-NTC |  | 0 |  | b act-NTC |  | 0 |  | b act-NTC |  | 0 |  |
| hspa5-NTC |  | 0 |  | hspa5-NTC |  | 0 |  | hspa5-NTC |  | 0 |  |

Crossing point (Cp) values of xbp1-s and b-actin after zebrafish embryos treated with 1-3 μg/ml tunicamycin or 5 μg/ml DMSO

| Experiment 1 | | | | Experiment 2 | | | | Experiment 3 | | | |
| --- | --- | --- | --- | --- | --- | --- | --- | --- | --- | --- | --- |
| Name | Cp | Standard | Status | Name | Cp | Standard | Status | Name | Cp | Standard | Status |
| xbp1s-1μg | 25.51 | 0 |  | xbp1s-1μg | 25.82 | 0 |  | xbp1s-1μg | 24.68 | 0 |  |
| xbp1s-1μg | 25.56 | 0 |  | xbp1s-1μg | 25.85 | 0 |  | xbp1s-1μg | 24.54 | 0 |  |
| xbp1s-1μg | 25.58 | 0 |  | xbp1s-2μg | 24.52 | 0 |  | xbp1s-2μg | 24.25 | 0 |  |
| xbp1s-2μg | 25.74 | 0 |  | xbp1s-2μg | 24.56 | 0 |  | xbp1s-2μg | 24.32 | 0 |  |
| xbp1s-2μg | 25.51 | 0 |  | xbp1s-3μg | 23.66 | 0 |  | xbp1s-3μg | 25.82 | 0 |  |
| xbp1s-2μg | 25.54 | 0 |  | xbp1s-3μg | 23.52 | 0 |  | xbp1s-3μg | 25.84 | 0 |  |
| xbp1s-3μg | 24.21 | 0 |  | xbp1s-DMSO | 26.54 | 0 |  | xbp1s-DMSO | 26.79 | 0 |  |
| xbp1s-3μg | 24.2 | 0 |  | xbp1s-DMSO | 26.55 | 0 |  | xbp1s-DMSO | 26.75 | 0 |  |
| xbp1s-3μg | 24.32 | 0 |  | b act-1μg | 21.72 | 0 |  | b act-1μg | 20.17 | 0 |  |
| xbp1s-DMSO | 26.74 | 0 |  | b act-1μg | 21.75 | 0 |  | b act-1μg | 20.54 | 0 |  |
| xbp1s-DMSO | 26.69 | 0 |  | b act-2μg | 21.19 | 0 |  | b act-2μg | 20.47 | 0 |  |
| xbp1s-DMSO | 26.74 | 0 |  | b act-2μg | 21.21 | 0 |  | b act-2μg | 20.5 | 0 |  |
| b act-1μg | 20.79 | 0 |  | b act-3μg | 19.87 | 0 |  | b act-3μg | 23.45 | 0 |  |
| b act-1μg | 20.98 | 0 |  | b act-3μg | 20.03 | 0 |  | b act-3μg | 23.53 | 0 |  |
| b act-1μg | 20.86 | 0 |  | b act-DMSO | 21.44 | 0 |  | b act-DMSO | 20.96 | 0 |  |
| b act-2μg | 21.54 | 0 |  | b act-DMSO | 21.52 | 0 |  | b act-DMSO | 21.17 | 0 |  |
| b act-2μg | 21.35 | 0 |  | b act-NTC |  | 0 |  | b act-NTC |  | 0 |  |
| b act-2μg | 21.29 | 0 |  | xbp1s-NTC |  | 0 |  | xbp1s-NTC |  | 0 |  |
| b act-3μg | 20.43 | 0 |  |  |  |  |  |  |  |  |  |
| b act-3μg | 20.44 | 0 |  |  |  |  |  |  |  |  |  |
| b act-3μg | 20.26 | 0 |  |  |  |  |  |  |  |  |  |
| b act-DMSO | 20.9 | 0 |  |  |  |  |  |  |  |  |  |
| b act-DMSO | 20.88 | 0 |  |  |  |  |  |  |  |  |  |
| b act-DMSO | 20.84 | 0 |  |  |  |  |  |  |  |  |  |
| b act-NTC |  | 0 |  |  |  |  |  |  |  |  |  |
| xbp1s-NTC |  | 0 |  |  |  |  |  |  |  |  |  |

| Experiment 4 | | | | Experiment 5 | | | | Experiment 6 | | | |
| --- | --- | --- | --- | --- | --- | --- | --- | --- | --- | --- | --- |
| Name | Cp | Standard | Status | Name | Cp | Standard | Status | Name | Cp | Standard | Status |
| xbp1s-1μg | 24.22 | 0 |  | xbp1s-1μg | 23.78 | 0 |  | xbp1s-1μg | 23.89 | 0 |  |
| xbp1s-1μg | 24.1 | 0 |  | xbp1s-1μg | 23.65 | 0 |  | xbp1s-1μg | 23.92 | 0 |  |
| xbp1s-2μg | 23.85 | 0 |  | xbp1s-2μg | 23.64 | 0 |  | xbp1s-2μg | 23.42 | 0 |  |
| xbp1s-2μg | 23.91 | 0 |  | xbp1s-2μg | 23.66 | 0 |  | xbp1s-2μg | 23.46 | 0 |  |
| xbp1s-3μg | 22.88 | 0 |  | xbp1s-3μg | 22.3 | 0 |  | xbp1s-3μg | 22.74 | 0 |  |
| xbp1s-3μg | 22.79 | 0 |  | xbp1s-3μg | 22.33 | 0 |  | xbp1s-3μg | 22.74 | 0 |  |
| xbp1s-DMSO | 25.43 | 0 |  | xbp1s-DMSO | 24.68 | 0 |  | xbp1s-DMSO | 25.55 | 0 |  |
| xbp1s-DMSO | 25.29 | 0 |  | xbp1s-DMSO | 24.81 | 0 |  | xbp1s-DMSO | 25.43 | 0 |  |
| b act-1μg | 19.34 | 0 |  | b act-1μg | 18.68 | 0 |  | b act-1μg | 19.07 | 0 |  |
| b act-1μg | 19.25 | 0 |  | b act-1μg | 18.72 | 0 |  | b act-1μg | 19.06 | 0 |  |
| b act-2μg | 19.45 | 0 |  | b act-2μg | 19.61 | 0 |  | b act-2μg | 18.64 | 0 |  |
| b act-2μg | 19.48 | 0 |  | b act-2μg | 19.57 | 0 |  | b act-2μg | 18.73 | 0 |  |
| b act-3μg | 19.34 | 0 |  | b act-3μg | 18.79 | 0 |  | b act-3μg | 19.01 | 0 |  |
| b act-3μg | 19.29 | 0 |  | b act-3μg | 18.84 | 0 |  | b act-3μg | 19.06 | 0 |  |
| b act-DMSO | 19.49 | 0 |  | b act-DMSO | 18.67 | 0 |  | b act-DMSO | 19.53 | 0 |  |
| b act-DMSO | 19.26 | 0 |  | b act-DMSO | 18.59 | 0 |  | b act-DMSO | 19.49 | 0 |  |
| b act-NTC |  | 0 |  | b act-NTC |  | 0 |  | b act-NTC |  | 0 |  |
| xbp1s-NTC |  | 0 |  | xbp1s-NTC |  | 0 |  | xbp1s-NTC |  | 0 |  |

Crossing point (Cp) values of ef1a and b-actin after zebrafish embryos treated with 1-3 μg/ml tunicamycin or 5 μg/ml DMSO

| Experiment 1 | | | | Experiment 2 | | | | Experiment 3 | | | |
| --- | --- | --- | --- | --- | --- | --- | --- | --- | --- | --- | --- |
| Name | Cp | Standard | Status | Name | Cp | Standard | Status | Name | Cp | Standard | Status |
| ef1a-1μg | 18.07 | 0 |  | ef1a-1μg | 18.63 | 0 |  | ef1a-1μg | 17.27 | 0 |  |
| ef1a-1μg | 17.94 | 0 |  | ef1a-1μg | 18.65 | 0 |  | ef1a-1μg | 17.3 | 0 |  |
| ef1a-1μg | 17.92 | 0 |  | ef1a-2μg | 17.73 | 0 |  | ef1a-2μg | 17.13 | 0 |  |
| ef1a-2μg | 18.53 | 0 |  | ef1a-2μg | 17.77 | 0 |  | ef1a-2μg | 17.16 | 0 |  |
| ef1a-2μg | 18.34 | 0 |  | ef1a-3μg | 17.42 | 0 |  | ef1a-3μg | 19.86 | 0 |  |
| ef1a-2μg | 18.43 | 0 |  | ef1a-3μg | 17.47 | 0 |  | ef1a-3μg | 19.94 | 0 |  |
| ef1a-3μg | 17.17 | 0 |  | ef1a-DMSO | 17.67 | 0 |  | ef1a-DMSO | 17.78 | 0 |  |
| ef1a-3μg | 17.2 | 0 |  | ef1a-DMSO | 17.78 | 0 |  | ef1a-DMSO | 17.98 | 0 |  |
| ef1a-3μg | 17.28 | 0 |  | b act-1μg | 21.72 | 0 |  | b act-1μg | 20.17 | 0 |  |
| ef1a-DMSO | 17.63 | 0 |  | b act-1μg | 21.75 | 0 |  | b act-1μg | 20.54 | 0 |  |
| ef1a-DMSO | 17.6 | 0 |  | b act-2μg | 21.19 | 0 |  | b act-2μg | 20.47 | 0 |  |
| ef1a-DMSO | 17.58 | 0 |  | b act-2μg | 21.21 | 0 |  | b act-2μg | 20.5 | 0 |  |
| b act-1μg | 20.79 | 0 |  | b act-3μg | 19.87 | 0 |  | b act-3μg | 23.45 | 0 |  |
| b act-1μg | 20.98 | 0 |  | b act-3μg | 20.03 | 0 |  | b act-3μg | 23.53 | 0 |  |
| b act-1μg | 20.86 | 0 |  | b act-DMSO | 21.44 | 0 |  | b act-DMSO | 20.96 | 0 |  |
| b act-2μg | 21.54 | 0 |  | b act-DMSO | 21.52 | 0 |  | b act-DMSO | 21.17 | 0 |  |
| b act-2μg | 21.35 | 0 |  | b act-NTC |  | 0 |  | b act-NTC |  | 0 |  |
| b act-2μg | 21.29 | 0 |  | ef1a-NTC |  | 0 |  | ef1a-NTC |  | 0 |  |
| b act-3μg | 20.43 | 0 |  |  |  |  |  |  |  |  |  |
| b act-3μg | 20.44 | 0 |  |  |  |  |  |  |  |  |  |
| b act-3μg | 20.26 | 0 |  |  |  |  |  |  |  |  |  |
| b act-DMSO | 20.9 | 0 |  |  |  |  |  |  |  |  |  |
| b act-DMSO | 20.88 | 0 |  |  |  |  |  |  |  |  |  |
| b act-DMSO | 20.84 | 0 |  |  |  |  |  |  |  |  |  |
| b act-NTC |  | 0 |  |  |  |  |  |  |  |  |  |
| ef1a-NTC |  | 0 |  |  |  |  |  |  |  |  |  |

| Experiment 4 | | | | Experiment 5 | | | | Experiment 6 | | | |
| --- | --- | --- | --- | --- | --- | --- | --- | --- | --- | --- | --- |
| Name | Cp | Standard | Status | Name | Cp | Standard | Status | Name | Cp | Standard | Status |
| ef1a-1μg | 17.1 | 0 |  | ef1a-1μg | 16.53 | 0 |  | ef1a-1μg | 16.77 | 0 |  |
| ef1a-1μg | 17.19 | 0 |  | ef1a-1μg | 16.62 | 0 |  | ef1a-1μg | 16.85 | 0 |  |
| ef1a-2μg | 17.15 | 0 |  | ef1a-2μg | 17.26 | 0 |  | ef1a-2μg | 16.47 | 0 |  |
| ef1a-2μg | 17.26 | 0 |  | ef1a-2μg | 17.24 | 0 |  | ef1a-2μg | 15.84 | 0 |  |
| ef1a-3μg | 16.79 | 0 |  | ef1a-3μg | 16.15 | 0 |  | ef1a-3μg | 16.64 | 0 |  |
| ef1a-3μg | 16.79 | 0 |  | ef1a-3μg | 16.22 | 0 |  | ef1a-3μg | 16.71 | 0 |  |
| ef1a-DMSO | 17.78 | 0 |  | ef1a-DMSO | 16.1 | 0 |  | ef1a-DMSO | 16.9 | 0 |  |
| ef1a-DMSO | 17.77 | 0 |  | ef1a-DMSO | 16.27 | 0 |  | ef1a-DMSO | 16.95 | 0 |  |
| b act-1μg | 19.34 | 0 |  | b act-1μg | 18.68 | 0 |  | b act-1μg | 19.07 | 0 |  |
| b act-1μg | 19.25 | 0 |  | b act-1μg | 18.72 | 0 |  | b act-1μg | 19.06 | 0 |  |
| b act-2μg | 19.45 | 0 |  | b act-2μg | 19.61 | 0 |  | b act-2μg | 18.64 | 0 |  |
| b act-2μg | 19.48 | 0 |  | b act-2μg | 19.57 | 0 |  | b act-2μg | 18.73 | 0 |  |
| b act-3μg | 19.34 | 0 |  | b act-3μg | 18.79 | 0 |  | b act-3μg | 19.01 | 0 |  |
| b act-3μg | 19.29 | 0 |  | b act-3μg | 18.84 | 0 |  | b act-3μg | 19.06 | 0 |  |
| b act-DMSO | 19.49 | 0 |  | b act-DMSO | 18.67 | 0 |  | b act-DMSO | 19.53 | 0 |  |
| b act-DMSO | 19.26 | 0 |  | b act-DMSO | 18.59 | 0 |  | b act-DMSO | 19.49 | 0 |  |
| b act-NTC |  | 0 |  | b act-NTC |  | 0 |  | b act-NTC |  | 0 |  |
| ef1a-NTC |  | 0 |  | ef1a-NTC | 38.53 | 0 | > - Late Cp call (last five cycles) has higher uncertainty | ef1a-NTC |  | 0 |  |

One-way analysis of variance and Tukey’s honestly significant different method (T-method) were conducted.
